# A lightweight alignment-aware DBNet for surgical instrument code detection

**DOI:** 10.1371/journal.pone.0355611

**Published:** 2026-08-07

**Authors:** Ke Yang, Yun Xue, Zhe Du, Shuchang Xu, Tian Tang, Zhifeng Qu

**Affiliations:** 1 School of Medical Technology and Engineering, Henan University of Science and Technology, Luoyang, China; 2 Henan Engineering Research Center of Digital Pathology and Artificial Intelligence Diagnosis, Luoyang, China; 3 The First Affiliated Hospital of Henan University of Science and Technology, Luoyang, China; Athens Medical Group, Psychiko Clinic, GREECE

## Abstract

Reliable detection of engraved surface codes on surgical instruments is essential for end-to-end traceability, yet remains challenging in practice because metallic reflection, motion blur, scale variation and weak textures often hinder stable localization. Here we present LA-DBNet, a lightweight detection framework built on DBNet for this task. The model uses MobileNetV4 with LiteFPN to reduce complexity while preserving multi-scale feature representations. To better capture the elongated structure and edge features of engraved codes, we introduce a Directional Edge Collaborative Alignment (DECA) module to improve cross-scale feature alignment, and embed an Efficient Channel Attention (ECA) mechanism in the high-resolution feature layer to enhance responses relevant to the target and suppress noise caused by reflections. We further incorporate a region-weighted consistency learning strategy during training to improve robustness to degraded samples. On our surgical instrument code dataset, LA-DBNet achieves an F1 of 95.8%, improving DBNet by 3.6 percentage points, while reducing parameters to 3.35 M and reaching 33.6 FPS. On ICDAR2015, it attains an F1 of 86.1%. These results show that LA-DBNet improves detection performance while substantially reducing model size and maintaining efficient inference in surgical instrument code detection.

## Introduction

End-to-end traceability of surgical instruments is fundamental to infection control and patient safety in modern healthcare systems [1]. Across decontamination, cleaning, packaging, sterilization, distribution and intraoperative use, each instrument must be uniquely identified and its handling history recorded to support quality assurance, error prevention and adverse-event management [2]. To enable management at the individual instrument level and full-process traceability in both regulatory and clinical practice, permanent direct part marking is typically applied to the instrument itself, most commonly in the form of engraved alphanumeric strings or two-dimensional codes linked to traceability records [3]. In real-world settings, however, reliable localization of these markings is hindered by specular reflection from metallic surfaces, highlight saturation, motion blur and scale variation. These effects are particularly detrimental to elongated characters, whose fine strokes and edge cues are prone to fragmentation or disappearance, thereby compromising stable detection and precise localization. In addition, the efficiency of code reading and the operator’s level of proficiency directly influence the cost and practicality of traceability workflows at the individual instrument level [4]. Developing robust methods for the detection and localization of alphanumeric codes on surgical instruments under challenging illumination and dynamic imaging conditions is therefore of substantial practical importance.

Text detection methods for industrial and natural scenes can generally be grouped into two main paradigms: regression-based and segmentation-based approaches [5]. Regression-based methods typically represent text instances as horizontal boxes, rotated boxes or quadrilaterals, and directly predict their geometry. CTPN, proposed by Tian et al. [6], employs fixed-width vertical anchors and combines CNN features with sequence modeling based on RNNs to connect text proposals, achieving strong performance on horizontal text. EAST, introduced by Zhou et al. [7], directly regresses text geometry using an end-to-end fully convolutional network, offering high inference efficiency and adaptability to multi-oriented text, but exhibiting limited robustness when dealing with elongated text, curved text and cluttered backgrounds. Segmentation-based methods, by contrast, describe text regions using pixel-wise probability maps and recover final instances through separation or aggregation strategies. PAN [8] improves speed substantially while maintaining competitive accuracy through a pixel aggregation mechanism, although there remains room for improvement in scenarios involving extreme aspect ratios. PSENet [9] uses progressive scale expansion to separate adjacent text instances, effectively alleviating adhesion between neighboring texts. FCENet [10] enhances curved-text modeling through Fourier contour representations. Mask R-CNN [11], as a general-purpose instance segmentation framework, provides strong region-level modeling capacity but is often associated with higher computational and deployment costs in text detection tasks involving high resolution images. Among segmentation-based methods, DBNet, proposed by Liao et al. [12], integrates the thresholding process into end-to-end optimization through differentiable binarization, achieving a favorable balance between detection accuracy and inference efficiency. It has therefore become a widely adopted baseline in practical applications. Its successor, DBNet++, further improves multi-scale text detection and robustness through adaptive scale fusion and related refinements [13].

Despite these advances, surgical instrument code detection remains particularly challenging for two main reasons. First, degradations such as reflection, highlight interference and blur destabilize character edges and stroke structures, making elongated engraved codes more susceptible to fragmentation, adhesion and missed detection. Second, many existing text detection networks remain computationally heavy and parameter-intensive, limiting inference efficiency and deployment feasibility. To address these limitations, we propose an improved DBNet-based framework for surgical instrument code detection that simultaneously enhances detection performance and reduces model complexity. The main contributions of this study are as follows.

(1) To overcome the large parameter count and limited inference efficiency of existing approaches to surgical instrument code detection, we develop LA-DBNet (Lightweight Alignment-aware DBNet), a lightweight detection framework built on DBNet. LA-DBNet adopts MobileNetV4 [14] as the backbone and integrates LiteFPN for lightweight multi-scale feature fusion, thereby reducing model size and accelerating inference while preserving as much structural detail as possible in high-resolution feature maps.
(2) To address the elongated morphology, weak texture, strong edge features, and frequent local misalignment encountered during cross-scale fusion, we design a Directional Edge Collaborative Alignment (DECA) module. By combining directional enhancement, discrepancy cues and recalibration with dual gates, DECA explicitly models the structural discrepancy between shallow detailed features and semantic features from deeper layers before fusion. This design improves the preservation of elongated character edges, weak texture regions and locally continuous structures. In addition, an Efficient Channel Attention (ECA) mechanism is introduced at the high-resolution feature level to further strengthen channel responses relevant to the target [15].
(3) To mitigate the instability caused by reflection, blur and low contrast, we introduce a Region-Weighted Consistency Learning (RWCL) strategy. By enforcing region-weighted consistency constraints between original and degraded views, RWCL concentrates supervision on the code body and its edge regions, thereby improving detection stability on severely degraded samples.

## Materials and methods

### Principle of DBNet

**Fig 1:**
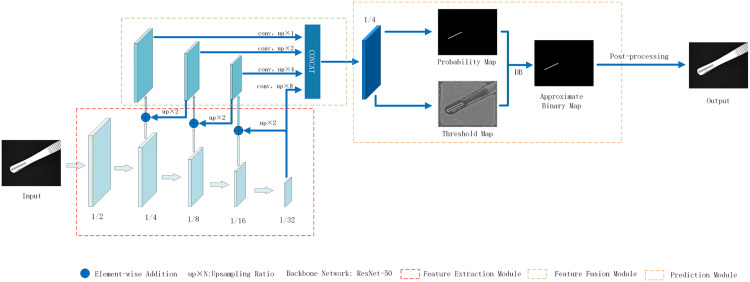
Architecture of the original DBNet. The original DBNet consists of three main parts: feature extraction, feature fusion, and prediction decoding. Multi-scale features extracted by the backbone are fused by a feature pyramid network (FPN), and the fused features are then fed into the DBHead to generate a probability map, a threshold map, and an approximate binary map for text detection.

DBNet is a segmentation-based scene text detection framework whose central innovation lies in the introduction of a differentiable binarization module, which incorporates the conventional thresholding step into network training and enables adaptive learning of binarization thresholds [16]. This design reduces the model’s sensitivity to manually specified fixed thresholds, thereby improving the stability of text region segmentation [17]. As shown in Fig 1, DBNet consists of three main components: feature extraction, feature fusion, and prediction decoding [18].

The feature extraction module typically uses ResNet [19] as the backbone to derive multi-scale representations. Through progressive downsampling, the network produces hierarchical feature maps with spatial resolutions of 1/2, 1/4, 1/8, 1/16 and 1/32 of the input image. These features are then fed into a Feature Pyramid Network (FPN) [20] for fusion. Following a top-down pathway, the FPN progressively upsamples high-level features and performs element-wise addition with shallow features at corresponding scales, thereby effectively integrating high-level semantic information with fine spatial detail. The fused features at each level are further refined using 3 × 3 convolutions, then upsampled to a unified spatial resolution of 1/4 of the input image, and finally concatenated along the channel dimension to produce the final fused feature map.

In the prediction decoding stage, the network generates a probability map and a threshold map in parallel from the fused features. The probability map indicates the likelihood that each pixel belongs to a text region, whereas the threshold map adaptively learns the binarization threshold for each spatial location. By jointly combining these two outputs, DBNet produces an approximate binary map, enabling fine-grained segmentation of text regions [21]. By embedding threshold learning into end-to-end optimization, this design improves segmentation performance in the presence of complex backgrounds and ambiguous text boundaries.

### Overall architecture of LA-DBNet

**Fig 2:**
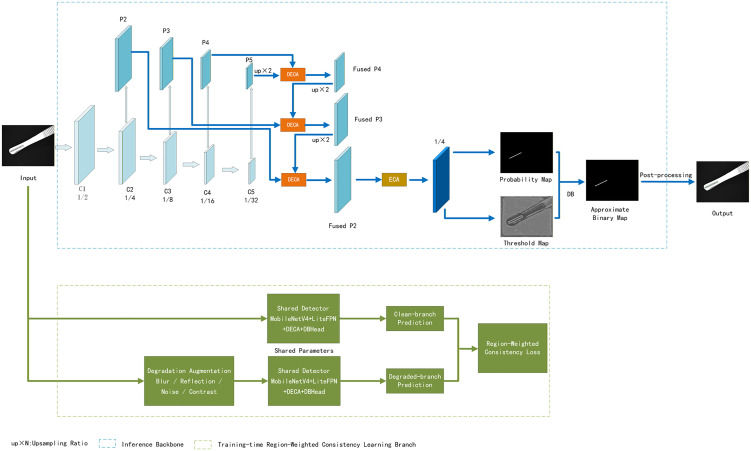
Architecture of LA-DBNet. LA-DBNet is built on a lightweight detection framework for surgical instrument code detection. The model uses MobileNetV4 as the backbone and LiteFPN for multi-scale feature fusion. DECA modules are introduced into the top-down fusion pathway to improve cross-scale alignment, and an efficient channel attention (ECA) module is applied to the highest-resolution output feature. During training, region-weighted consistency learning (RWCL) is further used to enhance robustness to degraded samples.

To balance detection accuracy and computational efficiency in surgical instrument code detection, we develop LA-DBNet, a lightweight framework built upon DBNet. Targeting the challenges commonly encountered in practical settings—including specular reflection, highlight interference, blur degradation and the tendency of elongated characters to fragment—LA-DBNet is designed as shown in Fig 2. The framework mainly consists of a lightweight backbone for feature extraction, a DECA-based cross-scale alignment and fusion module, an ECA-based output enhancement branch, and a region-weighted consistency learning module used only during training.

First, the lightweight backbone performs multi-scale feature extraction on the input image and feeds the resulting hierarchical features into the subsequent detection branches. During the top-down fusion process in LiteFPN, the DECA module is introduced to progressively align and enhance high-level semantic features with shallow detailed features, thereby improving the representation of elongated engraved strokes, character edges and local structural patterns. An ECA channel attention mechanism is then applied to the output feature map with the highest resolution to recalibrate channel responses, highlighting information relevant to code regions while suppressing interference caused by metallic reflection, bright highlights and background noise.

In the prediction stage, DBHead generates a probability map and a threshold map from the enhanced features, and computes an approximate binary map through differentiable binarization, ultimately yielding the location coordinates of the surface codes on surgical instruments. During training, a region-weighted consistency learning module is further introduced. By imposing region-weighted consistency constraints on the dual-branch predictions of the original and degraded images, this strategy improves the stability and robustness of the model under challenging degraded imaging conditions.

### Lightweight detection backbone

Surgical instrument code detection imposes stringent demands on both detection accuracy and inference efficiency. However, the original DBNet employs ResNet-50 as its backbone, resulting in a relatively large parameter count that is not well suited to deployment in resource-constrained settings. In addition, surface codes on surgical instruments are typically small in scale, composed of slender strokes, and highly susceptible to edge degradation caused by reflection and blur. The detection network must therefore provide strong fine-grained structural representation. To address these requirements, we adopt MobileNetV4 together with LiteFPN to construct a lightweight detection backbone, thereby reducing the number of parameters and the storage overhead while maintaining high inference efficiency.

#### Feature extraction.

As a lightweight backbone, MobileNetV4 enables hierarchical feature extraction with a relatively low parameter count and computational cost [22]. Compared with the original ResNet-50, its architecture is more carefully optimized to balance between accuracy and latency, making it better suited to detection tasks with strict inference-efficiency requirements. For surgical instrument code detection, shallow features retain higher spatial resolution and are therefore more effective at preserving character edges, stroke contours and local texture details, whereas deeper features encode richer semantic information that helps distinguish true code regions from highlights, reflective textures and background noise on metallic surfaces.

In our implementation, we adopt the small convolutional variant of MobileNetV4 as the backbone and select four intermediate-to-high-level feature maps for subsequent fusion. These features are then fed into LiteFPN to balance detailed representation at high resolution with semantic abstraction from deeper layers.

#### Feature fusion.

In the feature fusion stage, we employ LiteFPN as the neck network to aggregate the multi-scale features produced by the backbone. LiteFPN first applies 1 × 1 lateral convolutions to project features at different scales into a unified channel space, and then performs cross-scale fusion along a top-down pathway. The fused features at each level are subsequently refined and enhanced using depthwise separable convolutions. In our implementation, all feature maps are projected to a 128-channel space. Compared with the conventional FPN, LiteFPN [23] has a more compact architecture and enables cross-scale information interaction at lower computational cost, making it better suited to surgical instrument code detection, where both fine-grained structural representation and suppression of complex background interference are required.

On this basis, only the highest-resolution output feature P2 from LiteFPN is fed into the detection head. This P2 is not a purely shallow feature, but the output of the complete top-down feature pyramid fusion and has already aggregated deep semantic information. Therefore, the complete multi-scale pyramid is still used to progressively transfer deep semantics to the high-resolution feature level. Meanwhile, DECA improves structural alignment and edge collaboration during adjacent-scale fusion, enhancing the representation of elongated character edges, weak textures, and locally continuous structures in P2. This design avoids additional multi-scale upsampling and concatenation before the detection head, reducing computational burden while helping preserve engraved character edges and stroke structures.

### Directional edge collaborative alignment and attention enhancement mechanism

To address unstable responses at character edges, local structural misalignment during cross-scale fusion under severe degradations, and channel features that are insufficiently discriminative for the target, we introduce the DECA module into the top-down fusion pathway of LiteFPN and incorporate an ECA attention mechanism at the output stage. Together, these two components form a directional edge collaborative alignment and attention enhancement mechanism.

#### DECA module.

**Fig 3:**
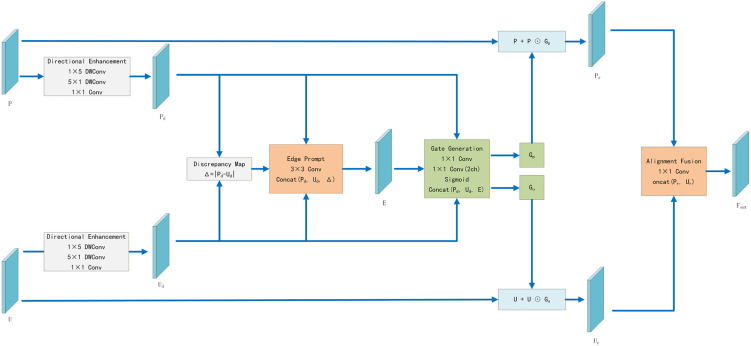
Structure of the DECA module. The directional edge collaborative alignment (DECA) module takes the high-resolution shallow feature and the upsampled high-level feature as inputs. It performs directional enhancement, difference prompting, dual-gated recalibration, and aligned fusion to improve structural matching across scales. This design helps preserve stroke continuity, edge responses, and local details of elongated code characters.

To better preserve elongated character structures, local edge information and code regions with weak texture during cross-scale feature fusion, we design a Directional Edge Collaborative Alignment (DECA) module, whose structure is illustrated in Fig 3.

This module is inserted at adjacent-scale fusion nodes along the top-down pathway of LiteFPN. It takes as input a shallow feature map with high resolution at the current scale and an upsampled high-level feature map from the upper scale, and processes them sequentially through directional enhancement, discrepancy prompting, recalibration with dual gates and alignment fusion, thereby improving structural correspondence during cross-scale integration. Compared with conventional feature concatenation or element-wise addition, DECA more effectively exploits cues from character edges and local structural discrepancies, thereby alleviating stroke fragmentation, boundary ambiguity and region adhesion under severe degradations.

Let the high-resolution shallow feature and the upsampled high-level feature be denoted by $P\in R^{C\times H\times W}$ and $U\in R^{C\times H\times W}$, respectively. To enhance the sensitivity of the features to edges and stroke structures with different orientations, directional enhancement mappings are first applied to both inputs, yielding the direction-aware features $P_{d}$ and $U_{d}$:


$$\begin{array}{c} \begin{array}{c} P_{d}=D(P) \end{array} \end{array}$$ (1)

$$\begin{array}{c} \begin{array}{c} U_{d}=D(U) \end{array} \end{array}$$ (2)

where D(·) denotes the directional enhancement operator. Because engraved characters on surgical instruments exhibit pronounced elongated morphology and directional anisotropy, we model local structures using two orthogonal depthwise convolution branches that operate along the horizontal and vertical directions, respectively. Their outputs are then combined through channel mixing to produce the final direction-enhanced representation, which can be formulated as:


$$\begin{array}{c} \begin{array}{c} D(\text{X})={Conv}_{\text{1}\times \text{1}}\left(Cat\left({DWConv}_{\text{1}\times \text{5}}(X),{DWConv}_{\text{5}\times \text{1}}(X)\right)\right) \end{array} \end{array}$$ (3)

Here, ${DWConv}_{\text{1}\times \text{5}}(\cdot )$ and ${DWConv}_{\text{5}\times \text{1}}(\cdot )$ denote depthwise convolutions applied along the horizontal and vertical directions, respectively, and Cat(·) denotes channel-wise concatenation. Without introducing substantial computational overhead, this design captures the directional characteristics and edge contours of elongated strokes more effectively.

After obtaining the direction-enhanced features, we further construct a discrepancy prompt map to explicitly characterize local differences between the two feature streams:


$$\begin{array}{c} \begin{array}{c} \Delta =\left|P_{d}-U_{d}\right| \end{array} \end{array}$$ (4)

here, Δ denotes the local response discrepancy between the direction-enhanced shallow feature and the upsampled high-level feature. Since both feature streams contain information related to code-region edges, stroke structures, and local textures, this discrepancy map highlights response inconsistencies between adjacent-scale features around weak boundaries and structurally continuous regions. In this study, the absolute difference is not an explicit geometric registration operation, but a lightweight feature-discrepancy cue used to assist subsequent gated recalibration and cross-scale fusion.

To further extract cues related to edges and local misalignment, $P_{d}$, $U_{d}$, and Δ are concatenated along the channel dimension and passed through an edge projection mapping to generate the edge-aware prompt feature E:


$$\begin{array}{c} \begin{array}{c} E=F_{edge}\left(Cat\left(P_{d},U_{d},D\right)\right) \end{array} \end{array}$$ (5)

where $F_{edge}(\cdot )$ denotes the edge projection function, and E is the resulting edge-aware prompt feature. By jointly integrating direction-enhanced responses and discrepancy information, this process provides a structural guidance for subsequent feature reweighting.

To regulate the contributions of shallow and high-level features at different spatial locations, we further introduce a dual-gating mechanism. Specifically, $P_{d}$, $U_{d}$, and E are concatenated and passed through a gating mapping to generate two spatial weight maps, $G_{p}$ and $G_{u}$:


$$\begin{array}{c} \begin{array}{c} \left[G_{p},G_{u}\right]=\sigma \left(F_{gate}\left(Cat\left(P_{d},U_{d},E\right)\right)\right) \end{array} \end{array}$$ (6)

where $F_{gate}(\cdot )$ denotes the gating mapping, and σ(·) is the Sigmoid activation function. The resulting maps $G_{p},G_{u}\in R^{\text{1}\times H\times W}$ serve as spatial gates for P and U, respectively. This design enables the two feature streams to be recalibrated independently according to their respective spatial response distributions, thereby better coordinating the fusion of fine-grained details and high-level semantic information.

On this basis, the input features are enhanced through a residual recalibration scheme:


$$\begin{array}{c} \begin{array}{c} P_{r}=P+P⨀G_{p} \end{array} \end{array}$$ (7)

$$\begin{array}{c} \begin{array}{c} U_{r}=U+U⨀G_{u} \end{array} \end{array}$$ (8)

where ⨀ denotes element-wise multiplication, and $P_{r}$ and $U_{r}$ represent the recalibrated shallow and high-level features, respectively. Compared with direct gating-based suppression, residual recalibration enhances responses in critical regions while preserving the main feature flow, thereby reducing the risk of losing useful information owing to overly aggressive gating.

Finally, the two recalibrated feature streams are concatenated along the channel dimension and passed through an alignment mapping to produce the fused output:


$$\begin{array}{c} \begin{array}{c} F_{out}=F_{align}\left(Cat\left(P_{r},U_{r}\right)\right) \end{array} \end{array}$$ (9)

where $F_{align}(\cdot )$ denotes the alignment fusion mapping, and $F_{out}$ is the final output feature of the DECA module. Through this process, the output feature preserves both high resolution detail and high-level semantic information, while achieving improved coordination in terms of directional structure, edge preservation and local alignment.

#### ECA.

Attention mechanisms can enhance a model’s representation of informative signals while suppressing irrelevant interference [24]. To reduce the adverse effects of responses associated with background textures, specular highlights and surface noise on the final detection results, we introduce an Efficient Channel Attention (ECA) module before the detection head, where it adaptively recalibrates the highest-resolution output feature along the channel dimension.

**Fig 4:**
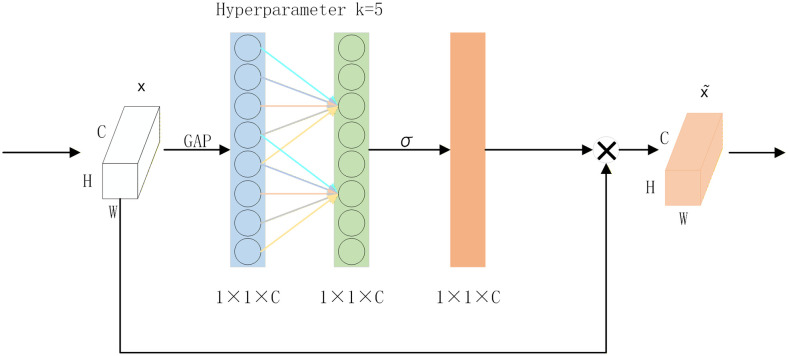
Structure of the ECA module. The efficient channel attention (ECA) module first applies global average pooling to extract channel-wise statistics, then uses a one-dimensional convolution to model local cross-channel interactions, and finally generates channel weights through a sigmoid function. The reweighted feature map enhances target-related responses while suppressing irrelevant background interference.

Specifically, ECA first extracts channel-wise statistical information through global average pooling, then captures local cross-channel interactions using a one-dimensional convolution, and finally generates channel weights through a Sigmoid activation to adaptively modulate the input features. The architecture of this module is illustrated in Fig 4 [25].

For surgical instrument code detection, this mechanism strengthens channel responses associated with character strokes, edge contours and textures of engraved regions, while suppressing interference introduced by metallic reflections, bright highlights and background noise. Because the feature map with the highest resolution directly supports subsequent pixel level detection, we apply ECA at this stage to enhance the discriminative power of the features for target regions with only minimal additional computational overhead.

### Region-weighted consistency learning strategy

When trained only with a conventional detection loss, the model primarily learns discriminative boundaries from normally captured samples; as a result, its predictions often become unstable in degraded scenarios characterized by strong highlights, blur and surface noise [26]. To improve robustness against such perturbations, we introduce a region-weighted consistency learning strategy. By imposing a consistency constraint between the original image and its degraded counterpart through two branches, this strategy encourages the network to maintain similar responses under different viewing conditions.

During training, the original input image *I* and its degraded augmented version $\tilde{I}$ are fed into a shared-weight detection network, producing the probability maps of the clean branch and the degraded branch, respectively:


$$\begin{array}{c} \begin{array}{c} P_{c}=f(I;\theta ) \end{array} \end{array}$$ (10)

$$\begin{array}{c} \begin{array}{c} P_{d}=f\left(\tilde{I};\theta \right) \end{array} \end{array}$$ (11)

where f(·;θ) denotes the shared-parameter detection network, and θ represents the network parameters. $P_{c}$ and $P_{d}$ correspond to the probability maps of text regions predicted from the original view and the degraded view, respectively. Because the two branches correspond to the same underlying image content and differ only in imaging quality, the network is expected to produce responses that are as consistent as possible within key regions. This dual-branch training scheme encourages the model to learn stable representations that are less sensitive to degradation, without introducing any additional overhead during inference.

If consistency constraints are imposed uniformly over the entire probability map, the loss will be dominated by the vast number of background pixels rather than the relatively small coded regions, thereby weakening supervision of the true targets. To address this issue, we introduce a region-weighting strategy that assigns larger weights to the main coded regions and smaller weights to background areas, so that the consistency constraint focuses more strongly on critical locations such as character bodies, edge contours and fine strokes.

The foreground mask (M) is generated by thresholding the high-response regions of the probability map produced by the clean branch. This mask is used solely to construct the region-weight map for the consistency regularization term and does not serve as a hard pseudo-label for supervision. The main detection loss remains supervised by manual annotations, which reduces the risk of erroneous optimization caused by inaccurate clean-branch predictions. Moreover, the background weight is kept nonzero, and the consistency loss is assigned a relatively small weight in the total loss. Thus, the influence of local inaccuracies in the foreground mask can be alleviated during optimization. The mask is defined as:


$$\begin{array}{c} \begin{array}{c} M_{i,j}=\left\{ \begin{array}{cc} \text{1},\; & P_{c}(i,j)\geq \tau \\ \text{0},\; & P_{c}(i,j)<\tau \end{array}\right. \end{array} \end{array}$$ (12)

where τ denotes the foreground mask threshold, and (i,j) represents the spatial coordinate. $M_{i,j}$ is a binary foreground mask of size H×W. On this basis, the region weight map *W* is defined as:


$$\begin{array}{c} \begin{array}{c} W=\alpha M+\beta (\text{1}-M) \end{array} \end{array}$$ (13)

where α and β denote the weighting coefficients for the foreground and background regions, respectively, with α>β.

The discrepancy between the probability maps predicted by the clean and degraded branches at corresponding locations is then measured using the Smooth L1 loss, and the resulting region-weighted consistency loss can be formulated as follows:


$$\begin{array}{c} L_{cons}=\frac{\text{1}}{HW}\sum_{i=\text{1}}^{H}\sum_{j=\text{1}}^{W}W_{i,j}\cdot SmoothL\text{1}\left(P_{c}(i,j),P_{d}(i,j)\right) \end{array}$$ (14)

where *H* and *W* denote the spatial dimensions of the probability map. By assigning greater weight to foreground regions while suppressing the contribution of background interference, this loss concentrates the consistency constraint on regions directly relevant to code localization, thereby alleviating unstable edge responses, the loss of fine strokes and reduced regional continuity under degraded conditions.

During overall optimization, the region-weighted consistency loss is jointly optimized with the main detection loss of DBNet. The total loss function is given by:


$$\begin{array}{c} \begin{array}{c} L=L_{db}+\lambda L_{cons} \end{array} \end{array}$$ (15)

where $L_{db}$ denotes the main detection loss, and λ is the weighting coefficient for the consistency loss. The main detection loss follows the original formulation of DBNet and consists of three components: the probability map loss, the threshold map loss and the approximate binary map loss:


$$\begin{array}{c} \begin{array}{c} L_{db}=L_{s}{+\lambda }_{t}L_{t}{+\lambda }_{b}L_{b} \end{array} \end{array}$$ (16)

where $L_{s}$, $L_{t}$, and $L_{b}$ denote the supervision terms for the probability map, threshold map, and approximate binary map, respectively, while $\lambda_{t}$ and $\lambda_{b}$ are the corresponding balancing coefficients.

## Results

### Datasets

Because no publicly available dataset currently exists for this task, we constructed our surgical instrument code dataset to evaluate the effectiveness and robustness of the proposed method in this specialized setting. To further evaluate the performance of the proposed method beyond the specialized surgical instrument dataset, we also conducted comparative experiments on the public ICDAR2015 benchmark.

ICDAR2015 is a widely used benchmark for scene text detection, containing 1,500 images, including 1,000 training images and 500 test images, all at a resolution of 1280 × 720. Captured using Google Glass, this dataset includes a substantial number of oriented, blurred and small-scale text instances, making it well suited for evaluating text detection performance in complex natural scenes [27].

Our surgical instrument code dataset contains 1,143 images, which were divided into 923 training images, 110 validation images and 110 test images, corresponding to an approximate ratio of 8:1:1. The dataset covers 55 categories of surgical instruments, including hemostatic forceps, needle holders, forceps, scissors, IUD hooks, uterine dilators, vaginal specula, dental mirrors and medicine cups. Each physical instrument was captured under different viewpoints and imaging conditions, with approximately 10–20 images collected for one instrument. All images were acquired under indoor illumination using a Hikvision industrial camera mounted on a custom acquisition platform. Because the focal length was not fixed, the resulting samples exhibit natural variation in imaging scale. The original image resolution is 2448 × 2048. Text regions were manually annotated using roLabelImg, with each complete code region labeled using the four vertices of a bounding rectangle.

**Fig 5:**
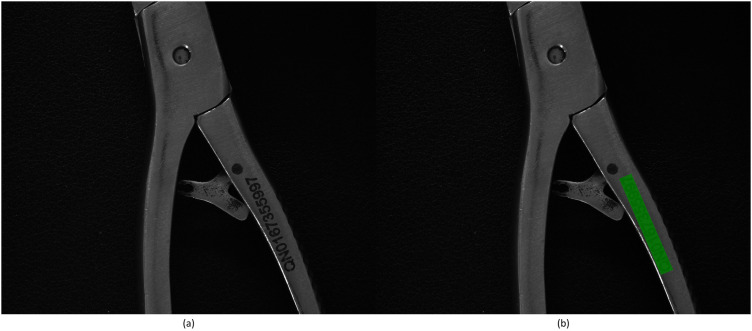
Examples of raw images and manual annotations. (a) Raw images of surgical instruments collected in real imaging conditions. (b) Corresponding manual annotations of code regions.

To avoid overly optimistic evaluation caused by highly similar samples appearing across different subsets, we adopted an instance-level split strategy, such that images of the same physical instrument instance appeared in only one of the training, validation or test sets. Meanwhile, during the instance-level split, we attempted to maintain approximately balanced category distributions among the training, validation, and test sets. In addition, we constructed a separate hard subset within the test set, accounting for approximately 25% of the test images, which mainly contains degraded samples affected by specular highlights, blur and low contrast. This subset was used to further evaluate detection performance under challenging imaging conditions. Fig 5 presents representative examples of raw images and their corresponding annotations from our surgical instrument code dataset.

### Experimental setup, implementation details and evaluation metrics

The hardware and software environment used in the experiments is summarized in Table 1. Model training was performed using the AdamW optimizer, with an initial learning rate of 0.0001 and a weight decay coefficient of 0.005. The models were trained for 300 epochs with a batch size of 1. The learning rate was scheduled using a warm-up strategy followed by polynomial decay. During training, data augmentation techniques including random flipping, slight rotation, contrast perturbation, Gaussian blur and scale jittering were applied. Unless otherwise stated, each model was trained and evaluated once using a fixed random seed. The reported inference speed was measured under the experimental environment listed in Table 1 and may be affected by factors such as the deep-learning framework, code implementation and optimization strategy, hardware configuration, batch size, and input resolution.

**Table 1:** Experimental environment configuration.

Configuration	Specification
Operating system	Windows 11
GPU	NVIDIA RTX 4090
CPU	Intel(R) Core(TM) i7-13700KF
Python	3.11
PyTorch	1.10.2
CUDA	11.3

Given the substantial differences between the self-collected surgical instrument code dataset and ICDAR2015 in terms of original image resolution, target-scale distribution, and scene characteristics, the input sizes were set to 2048 × 2048 and 1024 × 1024, respectively. For the self-collected dataset, the higher input resolution helps preserve the edges, strokes, and weak texture details of small engraved codes on surgical instruments. For ICDAR2015, text instances are relatively larger and differ substantially from the small engraved codes in the self-collected dataset. Therefore, 1024 × 1024 was adopted as the unified input size to preserve sufficient text details while avoiding excessive computational cost. During preprocessing, images were not directly stretched to 1024 × 1024. Instead, the original aspect ratio was preserved by resizing the longer side to 1024 pixels and scaling the shorter side proportionally, followed by padding to obtain a 1024 × 1024 input. The corresponding annotation coordinates were transformed using the same scaling factor and padding offset.

To ensure fair comparisons, all experiments conducted within each dataset adopted identical data splits, input resolutions, evaluation metrics, test hardware platforms and evaluation protocols. Because computational cost is closely tied to input resolution, the reported complexity results for different datasets are intended only for comparison within each dataset and are not used for direct cross-dataset comparison. All model variants proposed in this study were trained and tested under a unified training configuration. For models with publicly available implementations, their official training and post-processing settings were retained as much as possible. No selective task-specific tuning was applied to particular competing methods, in order to avoid subjective bias introduced by manual hyperparameter tuning.

For the region-weighted consistency learning strategy, a degraded-image branch was introduced during training in parallel with the original-image branch, with both branches sharing the same network parameters. The weight of the consistency loss was set to 0.10, the foreground mask threshold to 0.20, and the foreground and background region weights to 3.0 and 0.3, respectively. Degradation augmentations used in consistency training included blur, noise, contrast variation, highlight simulation and approximate JPEG compression, with the aim of improving robustness under complex imaging conditions.

Model performance was evaluated using Precision (P), Recall (R) and F1 score (F1). Results on both ICDAR2015 and our surgical instrument code dataset were calculated according to widely adopted public evaluation protocols for text detection.

Here, Precision represents the proportion of predicted positive samples that are truly positive, whereas Recall denotes the proportion of all true positive samples that are correctly detected. The F1 is the harmonic mean of Precision and Recall and is computed as follows:


$$\begin{array}{c} \begin{array}{c} P=\frac{TP}{TP+FP} \end{array} \end{array}$$ (17)

$$\begin{array}{c} \begin{array}{c} R=\frac{TP}{TP+FN} \end{array} \end{array}$$ (18)

$$\begin{array}{c} \begin{array}{c} F\text{1}=\frac{\text{2}PR}{P+R} \end{array} \end{array}$$ (19)

where TP denotes the number of true positives, FP the number of false positives, and FN the number of false negatives.

### Model complexity comparison

To evaluate the lightweight design of the proposed method, we compared the number of parameters, computational cost and model size of the original DBNet and LA-DBNet, as summarized in Table 2. It should be noted that all statistics reported in this section were measured on the model used at the inference stage only and therefore do not include the region-weighted consistency learning branch, which is introduced exclusively during training.

**Table 2:** Comparison of model size and complexity across different network architectures. The input resolution was 2048 × 2048.

Model	Parameters/ 10^6^	Computation/10^9^	Model size/ MB
DBNet	25.41	471.47	102
LA-DBNet	3.35	240.54	14.5

Specifically, LA-DBNet adopts MobileNetV4 as the backbone, uses LiteFPN for feature fusion, and incorporates the DECA cross-scale alignment module together with an ECA output enhancement branch. As shown in Table 2, compared with the baseline DBNet, LA-DBNet reduces the number of parameters from 25.41 M to 3.35 M (a decrease of approximately 86.8%), lowers the computational cost from 471.47 GFLOPs to 240.54 GFLOPs (a reduction of approximately 49.0%), and decreases model size from 102 MB to 14.5 MB (a reduction of approximately 85.8%). These results demonstrate that the proposed method substantially reduces overall model complexity.

### Ablation and comparative experiments

#### Ablation study on input resolution.

To analyze the effect of input resolution on detection performance and inference efficiency, we evaluated the model using input sizes of 1024 × 1024, 1280 × 1280, 1536 × 1536, and 2048 × 2048, while keeping the model architecture, training strategy, and evaluation protocol unchanged. The results are shown in Table 3. Since the model architecture remained unchanged, the number of parameters was 3.35 M for all input resolutions.

**Table 3:** Input-resolution ablation results on the self-collected surgical instrument code dataset. All experiments used the same model architecture, training strategy, and evaluation protocol. GPU FPS and CPU FPS were measured on the same hardware platform with a batch size of 1.

Input size	Parameters/ 10^6^	Computation/ 10^9^	GPU FPS	CPU FPS	Peak memory / MB	P (%)	R (%)	F1 (%)
1024 × 1024	3.35	60.13	128.1	2.92	298.58	97.0	88.2	92.4
1280 × 1280	3.35	93.96	77.2	2.45	457.91	95.2	90.9	93.1
1536 × 1536	3.35	135.3	51.7	2.09	632.92	97.1	91.8	94.4
2048 × 2048	3.35	240.54	33.6	1.06	1096.51	99.1	92.7	95.8

As shown in Table 3, Recall and F1 generally improved as the input resolution increased. When the input size increased from 1024 × 1024 to 2048 × 2048, F1 improved from 92.4% to 95.8%, indicating that higher-resolution inputs help preserve the edges, strokes, and weak texture details of small engraved codes on surgical instruments. Meanwhile, the computation increased from 60.13 GFLOPs to 240.54 GFLOPs, GPU FPS decreased from 128.1 to 33.6, CPU FPS decreased from 2.92 to 1.06, and peak memory increased from 298.58 MB to 1096.51 MB. These results show that higher input resolution introduces greater computational and memory overhead. Since this study mainly focuses on accurate detection of small engraved codes under challenging imaging conditions, and the 2048 × 2048 input size achieved the highest F1, it was adopted as the default input resolution for the self-collected dataset.

#### Ablation study.

To quantify the contribution of each proposed component to overall performance, we conducted an ablation study on our surgical instrument code dataset. The results are summarized in Table 4.

**Table 4:** Main ablation results on the self-collected surgical instrument code dataset. The input resolution was 2048 × 2048, and FPS was measured on the same hardware platform with a batch size of 1.

Model	Parameters/ 10^6^	Computation/ 10^9^	GPU FPS	P (%)	R (%)	F1 (%)
DBNet	25.41	471.47	18.5	99.0	86.4	92.2
M-DBNet	3.10	270.08	41	91.4	86.3	88.8
ML-DBNet	1.56	35.41	50.9	94.1	87.3	90.6
MLD-DBNet	3.35	240.5	33.8	98.0	89.0	93.3
MLDE(P2)-DBNet	3.35	240.54	33.7	98.1	90.0	93.8
LA-DBNet	3.35	240.54	33.6	99.1	92.7	95.8

As shown in Table 4, replacing the ResNet-50 backbone of DBNet with MobileNetV4 substantially reduces both the parameter count and computational cost. As a result, the inference speed of M-DBNet increases from 18.5 FPS to 41.0 FPS, although the F1 drops to 88.8%. This indicates that, while a lightweight backbone alone can markedly improve efficiency, it also leads to some loss in feature representation capacity. When the original FPN is further replaced with LiteFPN, ML-DBNet reduces the parameter count to 1.56 M and the computational cost to 35.41 GFLOPs, while the F1 recovers to 90.6%. This suggests that a lightweight neck architecture can preserve multi-scale feature fusion capability while substantially lowering model complexity.

On this basis, introducing DECA raises the F1 of MLD-DBNet from 90.6% to 93.3%, with Recall improving from 87.3% to 89.0%, indicating that cross-scale feature alignment and modeling of edge details are beneficial for this task. At the same time, the inclusion of DECA introduces additional computational overhead. Taken together, Tables 2 and 4 show that the proposed method achieves its lightweight advantage through overall optimization relative to the original DBNet. This advantage is reflected in parameter count, model size, and inference efficiency, rather than in the lowest computational cost of every intermediate variant. Adding ECA to MLD-DBNet further improves the F1 of MLDE(P2)-DBNet to 93.8%, while keeping the parameter count and inference speed essentially unchanged. This demonstrates that lightweight channel recalibration at the high-resolution feature level can improve responses relevant to the target at very low cost. Finally, after incorporating region-weighted consistency learning, LA-DBNet achieves 99.1% Precision, 92.7% Recall and an F1 of 95.8%. Compared with MLDE(P2)-DBNet, the F1 improves by a further 2.0 percentage points, showing that introducing region consistency constraints for degraded samples during training can further enhance detection performance without adding any extra branch during inference.

#### Comparison of lightweight backbones.

To evaluate the suitability of different lightweight backbones within the proposed framework, we conducted comparative experiments using ShuffleNetV2 [28], EfficientNet [29], MobileNetV3 [30] and MobileNetV4 as the backbone network, while keeping LiteFPN, DECA, ECA, RWCL and the detection head unchanged. The results are presented in Table 5.

**Table 5:** Comparison of different lightweight backbones on the self-collected surgical instrument code dataset. The input resolution was 2048 × 2048, and all other network modules and training settings were kept unchanged.

Backbone	Parameters/ 10^6^	Computation/ 10^9^	GPU FPS	P (%)	R (%)	F1 (%)
ShuffleNetV2	3.37	237.98	32.9	95.2	89.1	92.0
EfficientNet	4.96	253.67	30.4	96.2	90.9	93.5
MobileNetV3	2.95	228.44	32.5	95.9	85.4	90.4
MobileNetV4	3.35	240.54	33.6	**99.1**	**92.7**	**95.8**

As shown in Table 5, the choice of lightweight backbone has a clear impact on both detection performance and inference efficiency. ShuffleNetV2 and MobileNetV3 achieve relatively low parameter counts and fast inference speeds, but their Recall values are comparatively limited. In particular, MobileNetV3 attains a Recall of only 85.4%, indicating a higher tendency to miss code regions with weak texture, low contrast or blurred boundaries. EfficientNet reaches an F1 of 93.5%, outperforming the former two models in overall accuracy; however, this improvement comes at the cost of a larger parameter count and relatively slower inference. By contrast, MobileNetV4 delivers the highest Precision, Recall and F1 while maintaining only 3.35 M parameters and 33.6 FPS, demonstrating the most favorable overall balance between accuracy and efficiency.

#### Fine-grained ablation study of DECA.

To evaluate the effectiveness of each component in DECA, we conducted a fine-grained ablation study based on Full DECA, as shown in Table 6.

**Table 6:** Fine-grained ablation results of the DECA module on the self-collected surgical instrument code dataset. The input resolution was 2048 × 2048.

Model	P (%)	R (%)	F1 (%)
MLDE(P2)-DBNet (Full DECA)	**98.1**	90.0	**93.8**
without Directional Enhancement	96.9	86.4	91.4
3 × 3 DWConv instead of directional convolution	96.1	89.1	92.5
5 × 1 only	98.0	87.3	92.3
1 × 5 only	96.2	90.0	93.0
1 × 5 + 5 × 1 + 3 × 3	96.9	90.1	93.4
without Discrepancy Prompt	94.3	**90.9**	92.6
Cosine distance instead of absolute difference	97.1	90.0	93.4
Single Gate instead of Dual Gates	97.9	88.2	92.8

As shown in Table 6, Full DECA achieved the highest F1 of 93.8%. When Directional Enhancement was removed, F1 decreased to 91.4%, and Recall dropped from 90.0% to 86.4%, indicating that directional enhancement helps capture the edges and stroke structures of elongated engraved characters and reduces missed detections. Replacing directional convolution with 3 × 3 DWConv resulted in an F1 of 92.5%. Using only the 5 × 1 or 1 × 5 branch achieved F1 values of 92.3% and 93.0%, respectively, while adding an additional 3 × 3 branch resulted in an F1 of 93.4%, which was still lower than that of Full DECA. These results suggest that the dual-directional 1 × 5 and 5 × 1 modeling is more suitable for the elongated code structures in this task, and adding an extra 3 × 3 branch does not further improve performance.

Removing the Discrepancy Prompt decreased F1 to 92.6%, showing that the discrepancy cue helps alleviate local response inconsistencies during cross-scale fusion. Replacing absolute difference with cosine distance achieved an F1 of 93.4%, indicating that absolute difference is more suitable for capturing local response discrepancies in this task. Replacing Dual Gates with a Single Gate reduced F1 to 92.8%, suggesting that the dual-gate mechanism can more effectively recalibrate shallow detail features and high-level semantic features separately. These results demonstrate that directional enhancement, discrepancy prompt, absolute difference, and dual gates all contribute to DECA, with directional enhancement showing the most pronounced effect.

#### Comprehensive comparison of different fusion and enhancement configurations.

To isolate the effect of the DECA module on cross-scale feature fusion, we performed a module replacement study within the full model framework. Specifically, the fusion and alignment component at the top-down fusion nodes of LiteFPN was alternately implemented as direct addition (Add), attention-based fusion (Aff) and DECA, while all other settings were kept unchanged. The results are reported in Table 7.

**Table 7:** Comparison of different cross-scale fusion modules on the self-collected surgical instrument code dataset. The input resolution was 2048 × 2048, and FPS was measured on the same hardware platform with a batch size of 1.

Fusion strategy	Parameters/ 10^6^	Computation/ 10^9^	GPU FPS	P (%)	R (%)	F1 (%)
Add	1.56	35.44	49.1	97.0	87.2	91.9
Aff	1.61	38.42	42.2	94.9	85.5	90.0
DECA (LA-DBNet)	3.35	240.54	33.6	**99.1**	**92.7**	**95.8**

As shown in Table 7, different cross-scale fusion strategies exhibit marked differences in both detection performance and computational cost. Add has the simplest structure, the lowest computational overhead and the fastest inference speed, yet yields an F1 of only 91.9%, indicating that straightforward element-wise addition is insufficient to resolve the semantic discrepancy and spatial misalignment between features at different scales. Aff introduces a modest increase in parameters and computation, but its F1 declines to 90.0%, suggesting that conventional weighting based on attention does not effectively improve fusion quality and may instead introduce redundant responses that interfere with useful feature integration. By contrast, although DECA incurs a higher computational cost, it raises the F1 to 95.8% and delivers the most pronounced improvement in Recall, highlighting its superior ability to achieve cross-scale alignment while preserving edge detail.

#### Comparison of ECA placement at different feature levels.

To further determine the most effective placement of the ECA module, we compared two configurations on our surgical instrument code dataset: applying ECA only to the P2 feature level and applying it to all feature levels. The results are presented in Table 8.

**Table 8:** Comparison of different ECA insertion levels on the self-collected surgical instrument code dataset. The input resolution was 2048 × 2048, and FPS was measured on the same hardware platform with a batch size of 1.

Model	Parameters/ 10^6^	Computation/ 10^9^	GPU FPS	P (%)	R (%)	F1 (%)
MLDE (P2)-DBNet	3.35	240.54	33.7	98.1	**90.0**	**93.8**
MLDE (All)-DBNet	3.35	240.55	33.2	**98.9**	88.2	93.2

As shown in Table 8, when ECA is applied only to P2, the F1 increases from 93.2% to 93.8%, while Recall improves from 88.2% to 90.0%, with a slightly higher FPS as well. This indicates that, for the present task, concentrating attention on the high-resolution feature level is more effective. A likely reason is that the P2 feature map preserves richer local texture and edge information, which is particularly critical for recognizing small characters, elongated strokes and blurred boundaries.

#### Hyperparameter sensitivity analysis of RWCL.

To further justify the hyperparameter settings of the proposed region-weighted consistency learning (RWCL) strategy, we conducted a one-factor-at-a-time sensitivity analysis on the foreground-region selection threshold τ, the foreground consistency weight α, and the background consistency weight β based on the complete model. During the analysis, only one hyperparameter was varied at a time, while the others were fixed at their default values, namely τ = 0.20, α = 3, and β = 0.3. The results are summarized in Table 9.

**Table 9:** Sensitivity analysis of key RWCL hyperparameters on the self-collected surgical instrument code dataset. The input resolution was 2048 × 2048.

Parameter	Value	P (%)	R (%)	F1 (%)
without RWCL	—	98.1	90.0	93.8
τ	0.1	96.4	93.6	94.9
τ	0.15	99.0	91.8	95.3
τ	0.2	**99.1**	92.7	**95.8**
τ	0.25	94.6	**96.4**	95.5
τ	0.3	98.1	93.6	**95.8**
α	1	93.7	94.6	94.1
α	2	93.8	95.5	94.6
α	3	**99.1**	92.7	**95.8**
α	4	98.1	92.7	95.3
α	5	98.0	90.9	94.3
β	0.1	94.6	95.4	95.0
β	0.2	95.4	95.5	95.5
β	0.3	**99.1**	92.7	**95.8**
β	0.5	98.1	91.8	94.8
β	1	95.3	92.7	94.0

As shown in Table 9, after introducing RWCL, all tested parameter settings achieved higher F1 than the model without RWCL, which obtained an F1 of 93.8%. This indicates that RWCL provides a stable performance improvement. For the foreground threshold τ, both τ = 0.20 and τ = 0.30 achieved an F1 of 95.8%; however, τ = 0.20 produced higher Precision, suggesting a better balance between false-positive suppression and text-region coverage. For the foreground weight α, F1 increased as α increased from 1 to 3 and reached 95.8% at α = 3. Further increasing α led to a decrease in F1, indicating that an overly strong foreground consistency constraint may interfere with the optimization of the main detection objective. For the background weight β, the best F1 was obtained at β = 0.3, while further increasing the background weight reduced the performance, suggesting that excessive background consistency may weaken the learning of weak text and boundary regions.

Based on these results, τ = 0.20, α = 3, and β = 0.3 were selected as the default hyperparameter settings for RWCL. This configuration achieved a favorable balance among Precision, Recall, and F1, and also verifies that RWCL maintains stable performance within a reasonable parameter range.

### Comparison with different models

To validate the effectiveness of the proposed method, we compared LA-DBNet with several representative text detection models, including FCENet, Mask R-CNN, PANet, PSENet, EAST, DBNet++, DBNet, PP-OCRv4, and YOLOv11, on both our surgical instrument code dataset and the public ICDAR2015 benchmark. All models were evaluated on the same hardware platform. For competing methods relying on publicly available implementations, we retained their default training and post-processing settings as provided in the official releases, while ensuring comparison under a unified evaluation protocol.

The results on our surgical instrument code dataset are presented in Table 10.

**Table 10:** Comparison of different detection models on the self-collected surgical instrument code dataset. The input resolution was 2048 × 2048; GPU FPS and CPU FPS were measured on the same hardware platform with a batch size of 1.

Model	Parameters / 10^6^	Computation / 10^9^	GPU FPS	CPU FPS	Peak memory / MB	P (%)	R (%)	F1 (%)
YOLOv11	9.71	230.75	21.77	2.06	734.57	97.0	91.8	94.3
PP-OCRv4	3.57	52.04	6.75	0.16	1021.68	96.2	91.7	93.9
FCENet	26.26	415.35	10.2	0.94	1086.8	98.9	85.5	91.7
Mask R-CNN	43.97	582.12	11.2	0.67	1826.87	95.4	93.6	94.5
PANet	12.26	447.5	23.1	1.01	1576.44	97.8	84.6	90.7
PSENet	29.22	1332.87	11.4	0.74	3635.14	95.1	88.1	91.5
EAST	31.18	801.49	6.4	0.12	1423.66	97.9	88.9	93.2
DBNet++	26.03	626.5	13.2	0.69	2467.57	97.2	**93.7**	95.4
DBNet	25.41	471.47	18.5	0.75	1794.62	99.0	86.4	92.2
LA-DBNet	3.35	240.54	33.6	1.06	1096.51	**99.1**	92.7	**95.8**

LA-DBNet achieves 99.1% Precision, 92.7% Recall and an F1 of 95.8%, with the highest F1 among all compared methods, demonstrating its strong overall detection capability in this specialized application scenario. Compared with the baseline DBNet, LA-DBNet improves the F1 by 3.6 percentage points, while reducing the number of parameters from 25.41 M to 3.35 M, lowering the computational cost from 471.47 GFLOPs to 240.54 GFLOPs. Meanwhile, GPU FPS increased from 18.5 to 33.6, CPU FPS increased from 0.75 to 1.06, and peak GPU memory decreased from 1794.62 MB to 1096.51 MB. These results indicate that LA-DBNet improves detection performance while reducing model size, computational cost, and memory usage, and achieves favorable efficiency under both GPU and CPU inference settings.

The experimental results on the public ICDAR2015 dataset are summarized in Table 11.

**Table 11:** Detection results of different models on the public ICDAR2015 dataset. The input resolution was 1024 × 1024.

Model	Parameters/ 10^6^	Computation/ 10^9^	P (%)	R (%)	F1 (%)
FCENet	26.26	103.84	87.2	**83.6**	85.4
Mask R-CNN	43.97	145.53	86.2	77.9	81.9
PANet	12.26	111.87	87.6	77.6	82.3
PSENet	29.22	338.06	88.9	81.0	84.8
EAST	31.18	200.37	88.8	81.5	84.9
DBNet++	26.03	156.62	89.9	82.1	85.8
DBNet	25.41	117.87	88.5	78.4	83.2
LA-DBNet	3.35	60.13	**90.7**	82.0	**86.1**

On ICDAR2015, LA-DBNet achieves 90.7% Precision, 82.0% Recall and an F1 of 86.1%. It outperforms DBNet overall while maintaining a relatively low parameter count and computational cost. These results show that the proposed method performs well not only on our surgical instrument code dataset but also on the public ICDAR2015 benchmark, while maintaining clear advantages in model compactness and computational efficiency.

### Analysis of the effect of region-weighted consistency learning on challenging samples

To further examine the practical contribution of region-weighted consistency learning, we compared the detection performance of MLDE(P2)-DBNet, which does not incorporate consistency learning, with that of the full LA-DBNet model on both the entire test set and the hard subset. The results are summarized in Table 12.

**Table 12:** Effect of region-weighted consistency learning on detection performance for challenging samples. The input resolution was 2048 × 2048.

Model	F1 on the full test set (%)	F1 on the hard subset (%)
MLDE(P2)-DBNet	93.8	76.0
LA-DBNet	**95.8**	**83.1**

As shown in Table 12, MLDE(P2)-DBNet achieves an F1 of 93.8% on the full test set and 76.0% on the hard subset. After introducing consistency learning, LA-DBNet improves the F1 on the full test set to 95.8%, corresponding to a gain of 2.0 percentage points, while the F1 on the hard subset increases to 83.1%, representing a substantially larger gain of 7.1 percentage points. These results indicate that, under the present experimental setting, the incorporation of consistency learning enhances detection stability in the presence of severe degradations, with a particularly pronounced benefit on hard samples.

**Fig 6:**
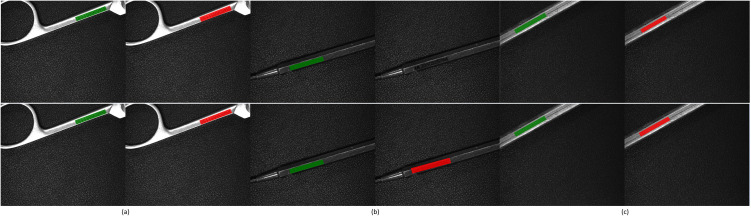
Comparison of detection results between MLDE(P2)-DBNet and LA-DBNet. Green boxes indicate ground truth annotations, and red boxes indicate predicted results. (a) Regular samples (b) Undetected samples (c) Broken samples.

Fig 6 presents the detection results of MLDE(P2)-DBNet and LA-DBNet on both regular and hard samples. The upper row shows the results of MLDE(P2)-DBNet, and the lower row shows those of LA-DBNet.

As illustrated in Fig 6, both models are able to detect the target text reasonably completely on regular samples, and the performance gap between them is comparatively small. In contrast, on hard samples, MLDE(P2)-DBNet is more prone to missed detections or incomplete localization. LA-DBNet, by comparison, is able to recover the target text regions more completely on the same samples and produces noticeably more stable detection results. This behavior can be attributed to the fact that hard samples are typically affected by specular reflection, blurred edges, insufficient contrast and poorly defined character structures, all of which make feature extraction and boundary localization more susceptible to noise. By introducing consistency learning, the model is encouraged during training to maintain consistent predictions under different views or perturbation conditions, thereby strengthening its focus on stable text regions and improving both feature representation and detection stability in challenging cases. Consequently, compared with the variant without consistency learning, LA-DBNet exhibits a much more pronounced improvement on hard samples.

### Model visualization and representative case analysis

To further analyze the behavior of the proposed method in challenging surgical instrument code detection scenarios, we visualized the feature responses of DECA, qualitative detection results under degraded conditions, and typical failure cases.

**Fig 7:**
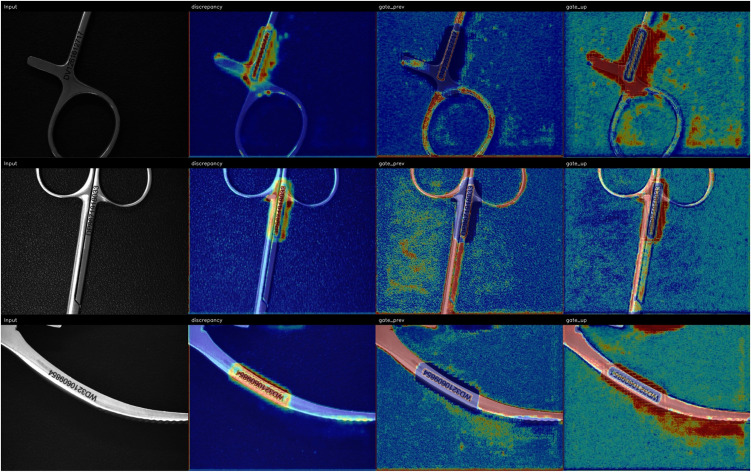
Feature-response visualization of the DECA module. Input denotes the original input image; discrepancy denotes the response map generated from the difference between the direction-enhanced shallow feature and the upsampled high-level feature; gate_prev and gate_up denote the gating response maps for shallow detail features and high-level semantic features, respectively. Stronger responses are mainly distributed around the code regions and their neighboring boundaries.

As shown in Fig 7, the discrepancy map generated by DECA shows relatively strong responses around the code regions and their neighboring boundaries, indicating that DECA can capture response differences related to text edges and local structures between adjacent-scale features. The gate_prev and gate_up maps reflect the gated responses of shallow detail features and high-level semantic features, respectively. Their different activation patterns around code regions and instrument structural edges suggest that the dual-gate mechanism adaptively recalibrates the two types of features.

**Fig 8:**
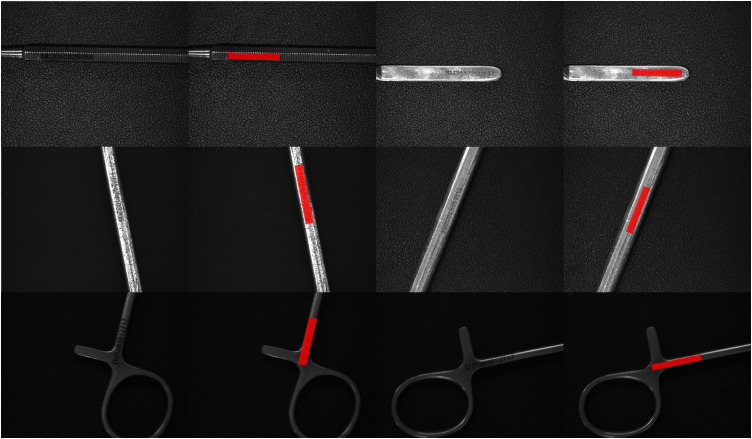
Qualitative detection results under challenging degraded conditions. The samples correspond to low contrast, reflection, wear, weak boundary, blur case I, and blur case II, respectively. Red boxes denote the predictions of LA-DBNet.

Fig 8 shows the detection results of LA-DBNet under challenging conditions, including reflection, blur, wear, weak boundaries, and low contrast. The proposed method can still localize code regions in most degraded samples, suggesting that DECA and region-weighted consistency learning improve the model’s adaptability to weak textures and complex imaging disturbances.

**Fig 9:**
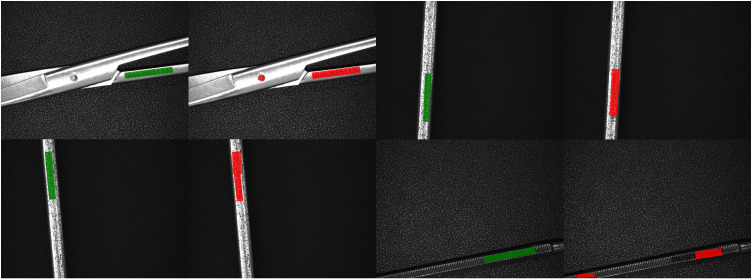
Typical failure cases. Green boxes denote ground-truth annotations, and red boxes denote model predictions. Under challenging conditions such as strong reflection, text-like structural edges, extremely weak engraving, or missing boundaries, the model may still produce false positives, missed detections, or localization shifts.

Fig 9 presents several typical failure cases. The green boxes denote ground-truth annotations, while the red boxes denote model predictions. When strong reflection, text-like structural edges, or extremely weak engraved codes appear on the instrument surface, the model may still produce false positives, missed detections, or localization shifts. These cases indicate that the proposed method remains limited under extreme low-contrast conditions, strong background interference, and severe boundary degradation. Future work may further incorporate reflection suppression, illumination normalization, and character-level contextual constraints to improve robustness under more challenging imaging conditions.

## Discussion

The results indicate that surgical instrument code detection requires not only a lightweight model but also effective preservation of fine structural information. The target regions in this task are typically elongated, weak in texture, and frequently affected by reflection, blur, and low contrast. In this setting, lightweight replacement alone can reduce computational cost but may also weaken the representation of local details needed for accurate localization. The present findings therefore support the use of a lightweight framework combined with task-oriented feature enhancement.

Ablation analysis further suggests that the performance improvement of LA-DBNet is mainly related to better cross-scale feature integration and stronger responses to fine-grained target cues. The contribution of DECA indicates that improved alignment between shallow detailed features and higher-level semantic features helps preserve the continuity of engraved code regions. The effectiveness of ECA at the high-resolution feature level likewise indicates the importance of enhancing local responses for this task. In addition, the gains provided by region-weighted consistency learning suggest that emphasizing supervision on code regions and their boundaries improves robustness under degraded imaging conditions, which is important for practical acquisition scenarios.

Several limitations remain in this study. First, the scale and acquisition diversity of the self-collected dataset are still limited, and further validation is needed across different imaging devices, illumination conditions, and more types of surgical instruments. Second, this study focuses mainly on code-region detection rather than a complete end-to-end code recognition and traceability pipeline. The regions detected by LA-DBNet can serve as inputs to downstream recognizers, such as CRNN, SVTR, or PP-OCR. However, because the current dataset mainly contains region-level annotations and complete character-level transcription labels have not yet been established, end-to-end recognition accuracy is not reported in this study. In addition, the degraded branch in region-weighted consistency learning is mainly constructed using simulated perturbations, which may not fully cover complex artifacts encountered during real image acquisition. Moreover, the reported results are based on a single training run for each model and therefore do not quantify the variability associated with random initialization; future work will include multiple independent runs with different random seeds. Finally, due to the limitation of available experimental platforms, deployment tests on Jetson devices, embedded GPUs, or industrial edge-computing platforms have not yet been conducted. The current CPU and GPU results therefore provide only a preliminary reference for deployment feasibility. Future work will expand the dataset and character-level annotations, integrate LA-DBNet with downstream recognition modules, and evaluate the complete system on realistic edge-computing platforms and practical acquisition scenarios.

## Conclusion

This study proposed LA-DBNet for surgical instrument code detection. By combining a lightweight architecture with task-oriented feature enhancement and consistency learning, the proposed method improved the detection of engraved code regions under challenging imaging conditions. On the in-house dataset, LA-DBNet achieved an F1 of 95.8%, outperforming the baseline DBNet while substantially reducing the number of parameters and increasing inference speed. The method also achieved favorable performance on the public ICDAR2015 benchmark. These findings indicate that LA-DBNet is a practical approach for surgical instrument code detection and has potential value for intelligent instrument traceability applications.
