# Supplementary material for: A lightweight alignment-aware DBNet for surgical instrument code detection
Source: PLoS One. 2026 Aug 7;21(8):e0355611. doi: 10.1371/journal.pone.0355611 (PMC13450737; doi:10.1371/journal.pone.0355611)
Supplement: Supplementary file 1 [file pone.0355611.s001.pdf]

This manuscript focuses on surgical instrument code detection and proposes a lightweight alignment-aware DBNet-based method. The study aims to address the difficulty of detecting engraved or printed codes on metallic surgical instruments under challenging imaging conditions, such as specular reflection, motion blur, low contrast, and weak object boundaries. Based on the DBNet framework, the authors introduce a lightweight backbone, a multi-scale feature fusion module, a directional edge collaborative alignment module, and a region-weighted consistency learning strategy.

Overall, the topic has practical significance, especially for surgical instrument traceability, sterilization management, and medical safety. The proposed framework is relatively complete, and the reported results show certain improvements over the baseline model on the self-collected dataset.

However, the current manuscript still has several important issues that need to be addressed. In particular, the justification of key hyperparameters is insufficient, the input-resolution settings are not clearly explained, the FPS evaluation protocol is ambiguous, the fairness of experimental comparisons needs further clarification, comparisons with recent methods are inadequate, and the reproducibility of the self-collected dataset remains unclear. Therefore, I recommend **major revision** before the manuscript can be considered for publication.

Major Comments:

1. Insufficient justification of key hyperparameters.

The proposed region-weighted consistency learning strategy involves several important hyperparameters, such as τ, α, and β. These parameters directly affect foreground-region selection, consistency-loss weighting, and training stability. However, the manuscript does not sufficiently explain how these parameters were determined.

The authors should clarify whether these values were selected based on validation-set tuning, empirical experience, or prior literature. A sensitivity analysis should also be provided to show how the model performance changes under different settings of τ, α, and β. In particular, τ appears to be an important threshold for foreground-region selection, and inappropriate selection may cause the model to focus on unreliable prediction regions or even introduce self-reinforcing errors.

2. The resizing strategy for ICDAR2015 needs clarification.

The manuscript states that, due to substantial differences between the self-collected surgical instrument code dataset and ICDAR2015 in terms of original image

resolution, target-scale distribution, and scene characteristics, the input images were resized to 2048 × 2048 and 1024 × 1024, respectively.

However, ICDAR2015 images usually have a non-square aspect ratio close to 1280 × 720. If these images are directly resized to 1024 × 1024, the original aspect ratio will be changed, which may distort the geometry, scale distribution, and shape of text instances.

Therefore, the authors should clearly specify the resizing strategy used for ICDAR2015. Was direct stretching to 1024 × 1024 applied, or was aspect-ratio-preserving resizing followed by padding used? If direct resizing was used, the authors should discuss its potential influence on text geometry and detection results. If padding was used, this should be explicitly stated in the experimental settings. The rationale for choosing 1024 × 1024 should also be provided.

3. The FPS evaluation protocol and result source should be clearly specified.

The manuscript reports FPS values, but the testing conditions are not sufficiently clear. Since the self-collected dataset and ICDAR2015 use different input resolutions, for example 2048 × 2048 and 1024 × 1024, the inference speed may vary substantially. Therefore, each FPS result should be clearly associated with its corresponding dataset, input resolution, and testing protocol.

4. An input-resolution ablation study is recommended.

The current experiments mainly use relatively high input resolutions. High-resolution input can preserve more fine-grained texture and boundary information of surgical instrument codes, which may improve detection performance. However, for a lightweight detection model, reducing the input resolution is also an important way to improve inference efficiency, reduce computational cost, and enhance deployability.

I suggest that the authors add an input-resolution ablation study, for example by comparing 2048 × 2048, 1536 × 1536, 1280 × 1280, and 1024 × 1024. The authors should report Precision, Recall, F1-score, FPS, GFLOPs, and memory usage under different input resolutions.

5. Deployment experiments on realistic platforms should be added.

The manuscript emphasizes lightweight design and practical application. However, the current speed evaluation appears to be mainly conducted on a high-performance GPU platform. For a practical surgical instrument code detection system, FPS on a high-end GPU alone is not sufficient to demonstrate real-world deployability.

The authors are encouraged to evaluate the proposed model on more realistic platforms, such as CPU, Jetson devices, embedded GPUs, or ordinary industrial workstations. In addition to FPS, memory consumption, model size, and end-to-end latency should also be reported. This would better support the claimed practical value of the proposed method.

6. Comparisons with more recent text detection methods should be included.

It is suggested that the authors investigate whether there are any recently proposed models with open-source implementations. If available, some of these newer models should be included for comparison to improve the persuasiveness of the experimental results.

8. Visualization results and failure-case analysis are insufficient.

The current visualization results are relatively limited. More qualitative results should be added, especially under challenging conditions such as severe specular reflection, motion blur, low contrast, worn engravings, curved metallic surfaces, occlusion, and small-scale code regions.

In addition, failure-case analysis should be provided. The authors should discuss under what conditions the proposed method still fails and explain the possible reasons. This is important for clarifying the limitations and application boundaries of the method.

Since the proposed DECA module is designed to enhance edge and alignment features, the authors are also encouraged to provide visual analyses related to this module, such as edge-response maps, attention maps, or feature activation maps, to more intuitively demonstrate its effectiveness for weak-boundary code detection.

9. Data and code availability should be strengthened.

The core experiments of this manuscript rely on a self-collected surgical instrument code dataset. To ensure reproducibility, the authors should provide a public data repository, DOI, or supplementary dataset archive. Ideally, the complete images, annotation files, training/validation/testing splits, difficult-subset definition, source code, and training configuration files should be made available.

If the dataset cannot be fully released due to privacy, institutional, or other restrictions, the authors should clearly explain these limitations and provide as much information as possible, such as anonymized examples, annotation format, dataset statistics, and reproducible experimental configurations. Otherwise, the reproducibility and credibility of the experimental conclusions may be weakened.

Minor Comments

1. The notation of evaluation metrics should be consistent throughout the manuscript. For example, if the F1-score is used as the main metric, the notation should be consistently written as "F1-score" or "F1" rather than using different symbols in different sections.
2. All result tables should clearly indicate the corresponding dataset, input resolution, and evaluation setting in the table caption or footnote. For example, captions such as "Results on the self-collected surgical instrument code dataset at 2048 × 2048 input resolution" or "Results on ICDAR2015 at 1024 × 1024 input resolution" would help avoid confusion.
