# Supplementary material for: A lightweight alignment-aware DBNet for surgical instrument code detection
Source: PLoS One. 2026 Aug 7;21(8):e0355611. doi: 10.1371/journal.pone.0355611 (PMC13450737; doi:10.1371/journal.pone.0355611)
Supplement: Supplementary file 2 [file pone.0355611.s003.docx]

Response to Reviewers

Manuscript number: PONE-D-26-18770

Title: A lightweight alignment-aware DBNet for surgical instrument code detection

Dear Editor and Reviewers,

We sincerely thank the editor and the two reviewers for their careful evaluation of our manuscript entitled “A lightweight alignment-aware DBNet for surgical instrument code detection” and for their valuable comments and suggestions. These comments have been very helpful in improving the experimental design, methodological description, and result analysis of our work. Following these suggestions, we have thoroughly revised the manuscript by adding additional experiments, clarifying the method description, and improving the discussion of the results. In this response letter, we provide point-by-point responses to the reviewers’ comments and describe the corresponding revisions made in the manuscript. We sincerely appreciate the constructive feedback from the editor and reviewers.

Our detailed responses are provided below.

**Reviewer #1**

**Comment 1:**

The manuscript mentions that only the highest-resolution output feature P2 from LiteFPN is retained as input to the detection head, yet it simultaneously inserts the DECA module into LiteFPN’s complete top-down fusion path for cross-scale alignment. If only P2 features are ultimately used, why is it necessary to construct a complete four-layer feature pyramid and perform DECA fusion across all adjacent scales? It is recommended to explicitly state which features are ultimately used for detection and to clarify the actual role of cross-scale fusion.

**Response to Comment 1:**

Thank you for this important comment. We agree that the relationship between the complete LiteFPN pathway and the final P2 feature should be clarified. In the revised manuscript, we have explicitly stated that only the highest-resolution output feature P2 is fed into the detection head. However, this P2 feature is not a purely shallow feature. It is generated after the complete top-down LiteFPN fusion process and therefore already incorporates semantic information propagated from deeper feature levels. The purpose of constructing the four-level feature pyramid is to allow high-level semantic cues to be progressively transferred to the high-resolution feature map, while the DECA modules improve cross-scale alignment between adjacent feature levels during this process. Therefore, although only P2 is used for final prediction, the complete feature pyramid and DECA-based fusion are still necessary for generating a semantically enriched and structurally aligned high-resolution feature map for detection.

**Comment 2:**

Directional enhancement relies solely on deep convolutions in two orthogonal directions (1×5 and 5×1), which fails to cover the oblique characters commonly found in surgical instrument codes. The manuscript does not explain why multi-directional convolutions or rotation-invariant feature extraction were not adopted, nor does it compare the performance differences among various directional combinations.

**Response to Comment 2:**

Thank you for this valuable suggestion. We agree that simply stating the use of two orthogonal directional convolution branches, i.e., 1×5 and 5×1, is not sufficient. Therefore, in the revised manuscript, we added a fine-grained ablation study on the directional enhancement component of DECA to compare the effects of different directional modeling strategies.

Specifically, we compared several configurations, including removing Directional Enhancement, replacing directional convolution with 3×3 DWConv, using only the 5×1 branch, using only the 1×5 branch, and adding an extra 3×3 branch to the 1×5 and 5×1 branches. The results show that removing Directional Enhancement reduced F1 from 93.8% to 91.4%, and Recall from 90.0% to 86.4%, indicating that directional enhancement plays an important role in reducing missed detections. Using only the 5×1 or 1×5 branch achieved F1 values of 92.3% and 93.0%, respectively. Replacing directional convolution with 3×3 DWConv resulted in an F1 of 92.5%. Adding an additional 3×3 branch to the 1×5 and 5×1 branches achieved an F1 of 93.4%, which was still lower than that of Full DECA, i.e., 93.8%.

These results indicate that the two orthogonal 1×5 and 5×1 branches can effectively model elongated strokes and edge continuity in engraved surgical instrument codes. Although this design is not an explicit rotation-invariant feature extraction method and cannot fully cover all oblique character patterns, it achieved better overall performance in this task than single-direction branches, standard 3×3 convolution, or the additional 3×3-branch configuration. Considering the lightweight design and inference efficiency requirements of the proposed model, we retained the 1×5 + 5×1 dual-direction design as the directional enhancement module. The corresponding results and analysis have been added to the fine-grained DECA ablation section of the revised manuscript.

**Comment 3:**

The difference map Δ directly uses the absolute difference of feature values. This operation can only characterize pixel-level intensity differences and cannot effectively capture spatial misalignment or semantic differences between cross-scale features. The manuscript does not demonstrate that the absolute difference is the optimal difference metric, nor does it compare the performance of other metrics such as cosine similarity or Euclidean distance.

**Response to Comment 3:**

Thank you for this insightful comment. We agree that the role and limitation of the absolute-difference operation should be more clearly explained. In the revised manuscript, we have clarified that the discrepancy map Δ is not computed from raw image pixel intensities. Instead, it is calculated from the direction-enhanced shallow feature and the direction-enhanced upsampled high-level feature after they have been projected to the same spatial scale and channel space. Therefore, Δ represents a local feature-response discrepancy rather than a raw pixel-intensity difference.

After convolutional encoding and directional enhancement, the feature responses contain information related to code-region edges, strokes, local textures, and semantic discrimination. Thus, the absolute-difference operation is used as a lightweight cue to highlight local response inconsistencies between shallow detail features and high-level semantic features, especially around weak boundaries and discontinuous engraved strokes. We have also clarified that this operation is not intended to serve as an explicit geometric registration module for estimating large spatial displacement. Instead, it provides a local discrepancy prompt, which is further processed together with the direction-enhanced features through an edge projection mapping. This enables the network to learn context-aware structural cues related to boundary misalignment, weak edges, and cross-scale response inconsistency.

To further justify the choice of the discrepancy metric, we added a fine-grained ablation experiment in the revised manuscript. Specifically, we replaced the absolute-difference operation with a cosine-distance-based alternative. The results show that the full DECA using absolute difference achieves an F1 score of 93.8%, whereas replacing it with cosine distance decreases the F1 score to 93.4%. This indicates that, under the present experimental setting, the position-wise absolute difference is slightly more effective for preserving response-magnitude differences around local boundaries and weak-texture regions. In contrast, cosine distance mainly focuses on feature-direction similarity and may weaken amplitude information related to edge-strength variation and local structural discontinuity.

We have revised the manuscript accordingly. We also avoided claiming that absolute difference is universally optimal. Instead, we state that it provides a favorable balance between detection performance and computational cost for the present surgical instrument code detection task.

**Comment 4:**

Why does the manuscript require two independent spatial gates to weight shallow and deep features separately? Why not adopt a simpler single-gate mechanism to control the fusion ratio between the two? There is a lack of ablation experiments to validate the necessity of the dual-gate design.

**Response to Comment 4:**

Thank you for this helpful suggestion. In the revised manuscript, we added a fine-grained ablation experiment by replacing the dual-gate design in DECA with a single-gate mechanism. The results show that the full DECA achieves an F1 score of 93.8%, whereas the single-gate variant decreases the F1 score to 92.8%. This confirms that independently recalibrating shallow detail features and upsampled high-level semantic features is beneficial for this task. The shallow and deep features have different response characteristics: shallow features preserve fine-grained edges and stroke details, while high-level features provide stronger semantic discrimination. A single gate imposes a coupled fusion ratio on both streams, whereas dual gates allow the two feature streams to be adjusted independently at each spatial location. Therefore, the dual-gate design provides more flexible feature recalibration and improves cross-scale fusion quality.

**Comment 5:**

The foreground mask in the manuscript is generated based on the probability map of the clean branch, and the threshold τ = 0.2 is an empirical value. The authors did not conduct a hyperparameter sensitivity analysis, so they cannot prove the universality of this threshold. Errors in the predictions of some clean branches can lead to an inaccurate foreground mask, which in turn causes the consistency loss to generate erroneous supervision signals.

**Response to Comment 5:**

Thank you for pointing this out. In the revised manuscript, we added a hyperparameter sensitivity analysis for the region-weighted consistency learning strategy. Specifically, we varied the foreground threshold τ, the foreground weight α, and the background weight β while keeping the other parameters fixed. The results show that τ = 0.20, α = 3, and β = 0.3 provide a favorable balance between Precision, Recall, and F1 score. For τ, the model maintains stable performance within a reasonable range, and τ = 0.20 achieves a good balance between text-region coverage and false-positive suppression.

We also clarified that the mask generated from the clean branch is not used as a hard pseudo-label to replace ground-truth supervision. Instead, it is used only to construct a region-weight map for the consistency loss. The main detection loss is still supervised by manual annotations. In addition, the background weight is not set to zero, and the consistency loss is assigned a relatively small coefficient in the total loss. These designs reduce the potential negative influence of inaccurate clean-branch predictions and prevent erroneous foreground masks from dominating the training process.

**Comment 6:**

The manuscript does not compare the proposed method with the latest lightweight text detection models, such as DBNet++, the MobileNetV3 backbone, YOLOv8/YOLOv9-based models, or the detection components of PP-OCRv3/v4. These models may offer advantages in terms of accuracy and speed.

**Response to Comment 6:**

Thank you for this constructive suggestion. We agree that comparisons with more recent and representative lightweight text detection methods can further strengthen the persuasiveness of the experimental results. In the original manuscript, we had already included comparisons with DBNet++ and different lightweight backbones, including ShuffleNetV2, EfficientNet, MobileNetV3, and MobileNetV4. Following the reviewer’s suggestion, we further expanded the comparison in the revised manuscript by adding PP-OCRv4 and a YOLO-series detector on the self-collected surgical instrument code dataset.

All compared methods were evaluated under the same data split, input resolution, evaluation metrics, and hardware platform. The experimental results show that LA-DBNet achieves the highest F1 score on the self-collected dataset, while maintaining a smaller model size and favorable inference efficiency compared with most conventional text detection methods. These additional comparisons further validate the effectiveness and lightweight advantage of the proposed method for surgical instrument code detection.

**Comment 7:**

The manuscript’s ablation experiments only verify the overall contributions of major modules and do not perform fine-grained ablation on key components within the modules. For example, what are the individual contributions of the direction enhancement, difference prompt, and dual-gating components within the DECA module? How much would removing any one of these components affect performance?

**Response to Comment 7:**

Thank you for this constructive comment. We agree that the ablation experiments in the original manuscript mainly verified the overall contribution of major modules, while the individual effects of key components within DECA were not sufficiently analyzed. Following the reviewer’s suggestion, we added a fine-grained ablation study of DECA in the revised manuscript, separately evaluating the effects of Directional Enhancement, Discrepancy Prompt, the discrepancy measurement strategy, and Dual Gates.

The results show that Full DECA achieved the highest F1 of 93.8%. Removing Directional Enhancement reduced F1 to 91.4%, indicating that directional enhancement helps capture the edges and stroke structures of elongated engraved characters. Removing the Discrepancy Prompt reduced F1 to 92.6%, and Precision decreased from 98.1% to 94.3%, suggesting that the discrepancy cue helps alleviate local response inconsistencies during cross-scale fusion and suppress false positives. Replacing absolute difference with cosine distance resulted in an F1 of 93.4%, slightly lower than that of Full DECA, indicating that absolute difference is more suitable for capturing local response discrepancies between adjacent-scale features in this task. Replacing Dual Gates with a Single Gate decreased F1 to 92.8%, showing that the dual-gate mechanism can more effectively recalibrate shallow detail features and high-level semantic features separately.

These results further validate the effectiveness of the key components within DECA. Among them, removing Directional Enhancement caused the most pronounced decrease in F1 and Recall, indicating that directional enhancement is particularly important for detecting elongated engraved codes. The corresponding experiments and analysis have been added to the fine-grained DECA ablation section of the revised manuscript.

**Comment 8:**

The manuscript mentions that the dataset is split at the instance level, but does not specify the exact splitting criteria or process. For example, is the split based on instrument instances or categories? How many images are there per instance? Does the splitting ensure category balance between the training and test sets?

**Response to Comment 8:**

Thank you for this careful comment. We have further clarified the dataset splitting strategy in the revised manuscript. The split was performed at the physical instrument instance level rather than at the image level. In our dataset, each physical instrument was captured under different viewpoints and imaging conditions, with approximately 10–20 images collected for one instrument. All images from the same physical instrument were assigned exclusively to one subset, so that visually similar images of the same instrument would not appear across the training, validation, and test sets. Meanwhile, we attempted to maintain approximately balanced category distributions among the three subsets. This clarification has been added to the dataset description section of the revised manuscript.

**Comment 9:**

The manuscript states that the default training and post-processing settings from the official implementation were retained, but no model-specific tuning was performed for the surgical instrument code dataset. Since LA-DBNet was specifically designed for this task, is this comparison method fair to other models?

**Response to Comment 9:**

Thank you for raising this important concern. We agree that the fairness of the comparative experiments should be further clarified. In the original manuscript, we had already stated that all compared methods were evaluated using the same training, validation, and test splits, as well as the same input resolution, evaluation metrics, and hardware platform within each dataset. For competing methods with publicly available implementations, we retained their official training and post-processing settings as much as possible to ensure reproducibility and to avoid subjective bias introduced by manual hyperparameter tuning.

Following the reviewer’s suggestion, we further clarified in the revised manuscript that no selective task-specific tuning was applied to particular competing methods. All methods were evaluated under a unified experimental protocol. The purpose of this setting was not to exhaustively search for the optimal hyperparameter combination for each model on this dataset, but to compare the overall performance of different models under consistent, public, and reproducible conditions. This additional clarification helps further support the fairness of the comparative evaluation.

**Comment 10:**

The manuscript only implements surgical instrument code detection and does not include a character recognition module, whereas an actual traceability system requires a complete detection and recognition pipeline. The authors do not address the compatibility of LA-DBNet with existing recognition models nor provide end-to-end recognition accuracy results, making it impossible to assess its practical application value.

**Response to Comment 10:**

Thank you for this important comment. We agree that a complete traceability system should include both code-region detection and downstream character recognition. The present study focuses on the detection stage, because stable localization of weakly engraved code regions is a prerequisite for reliable recognition in surgical instrument traceability. In the revised manuscript, we clarified the scope of this work and discussed the compatibility of LA-DBNet with existing recognition models. LA-DBNet outputs the detected code-region coordinates, which can be used to crop and rectify code regions before feeding them into recognition models such as CRNN-, SVTR-, or PP-OCR-based recognizers. However, the current dataset was annotated at the code-region level and does not yet contain complete character-level transcription labels for all samples. Therefore, we did not report end-to-end recognition accuracy in this study. We have added this point as a limitation and clarified that future work will integrate LA-DBNet with downstream recognition modules and construct character-level annotations to evaluate complete end-to-end traceability performance.

**Reviewer #2**

**Major Comment 1:**

Insufficient justification of key hyperparameters. The proposed region-weighted consistency learning strategy involves several important hyperparameters, such as τ, α, and β. These parameters directly affect foreground-region selection, consistency-loss weighting, and training stability. However, the manuscript does not sufficiently explain how these parameters were determined. The authors should clarify whether these values were selected based on validation set tuning, empirical experience, or prior literature. A sensitivity analysis should also be provided to show how the model performance changes under different settings of τ, α, and β. In particular, τ appears to be an important threshold for foreground-region selection, and inappropriate selection may cause the model to focus on unreliable prediction regions or even introduce self-reinforcing errors.

**Response to Major Comment 1:**

Thank you for this valuable comment. In the revised manuscript, we added a hyperparameter sensitivity analysis for τ, α, and β. A one-factor-at-a-time strategy was used: only one parameter was changed each time, while the other two were kept at their default values. The results show that τ = 0.20, α = 3, and β = 0.3 provide a favorable balance among Precision, Recall, and F1 score. The analysis also shows that too small a τ may introduce excessive background regions into the consistency constraint, while too large a τ may exclude weakly activated code regions. Similarly, a moderate foreground consistency weight improves robustness, whereas an overly large α or β may interfere with the supervised detection objective. Based on these observations, τ = 0.20, α = 3, and β = 0.3 were adopted as the default settings.

**Major Comment 2:**

The resizing strategy for ICDAR2015 needs clarification. The manuscript states that, due to substantial differences between the self-collected surgical instrument code dataset and ICDAR2015 in terms of original image resolution, target-scale distribution, and scene characteristics, the input images were resized to 2048 × 2048 and 1024 × 1024, respectively. However, ICDAR2015 images usually have a non-square aspect ratio close to 1280 × 720. If these images are directly resized to 1024 × 1024, the original aspect ratio will be changed, which may distort the geometry, scale distribution, and shape of text instances. Therefore, the authors should clearly specify the resizing strategy used for ICDAR2015. Was direct stretching to 1024 × 1024 applied, or was aspect-ratio-preserving resizing followed by padding used? If direct resizing was used, the authors should discuss its potential influence on text geometry and detection results. If padding was used, this should be explicitly stated in the experimental settings. The rationale for choosing 1024 × 1024 should also be provided.

**Response to Major Comment 2:**

Thank you for pointing out this important issue. We agree that ICDAR2015 images usually have non-square aspect ratios, and directly stretching them to 1024 × 1024 may distort the geometry and scale distribution of text instances. Therefore, in the revised manuscript, we further clarified the preprocessing strategy used for ICDAR2015.

Specifically, for ICDAR2015, images were not directly stretched to 1024 × 1024. Instead, the original aspect ratio was preserved during preprocessing. The longer side of each image was first resized to 1024 pixels, while the shorter side was scaled proportionally. Padding was then applied to obtain a 1024 × 1024 input image. The corresponding text annotation coordinates were also transformed using the same scaling factor and padding offset. Therefore, this preprocessing strategy avoids text-geometry distortion caused by direct stretching.

The input size of 1024 × 1024 was selected mainly because ICDAR2015 differs from our self-collected surgical instrument code dataset in terms of original resolution and relative text scale. Compared with the small and weak-textured engraved codes in our self-collected dataset, text instances in ICDAR2015 have a relatively larger scale, so the higher input resolution of 2048 × 2048 is not necessary. The 1024 × 1024 input size preserves sufficient text details for ICDAR2015 while avoiding unnecessary computational overhead for this dataset. We have added these clarifications regarding the preprocessing strategy and the rationale for input-size selection in the experimental settings section of the revised manuscript.

**Major Comment 3:**

The FPS evaluation protocol and result source should be clearly specified. The manuscript reports FPS values, but the testing conditions are not sufficiently clear. Since the self-collected dataset and ICDAR2015 use different input resolutions, for example 2048 × 2048 and 1024 × 1024, the inference speed may vary substantially. Therefore, each FPS result should be clearly associated with its corresponding dataset, input resolution, and testing protocol.

**Response to Major Comment 3:**

Thank you for this careful suggestion. We agree that when different input resolutions are used for different datasets, each FPS result should be clearly associated with its corresponding dataset, input size, and testing protocol. In this study, the reported FPS values were measured on the self-collected surgical instrument code dataset at an input resolution of 2048 × 2048. ICDAR2015 was mainly used to evaluate the generalization ability of the proposed method on a public scene text detection benchmark, and FPS results on ICDAR2015 were not separately reported.

Following the reviewer’s suggestion, we revised the captions or footnotes of all result tables containing FPS values to explicitly indicate the corresponding dataset, input resolution, and testing setting. Specifically, FPS was measured on the same hardware platform with a batch size of 1.

**Major Comment 4:**

An input-resolution ablation study is recommended. The current experiments mainly use relatively high input resolutions. High-resolution input can preserve more fine-grained texture and boundary information of surgical instrument codes, which may improve detection performance. However, for a lightweight detection model, reducing the input resolution is also an important way to improve inference efficiency, reduce computational cost, and enhance deployability. I suggest that the authors add an input-resolution ablation study, for example by comparing 2048 × 2048, 1536 × 1536, 1280 × 1280, and 1024 × 1024. The authors should report Precision, Recall, F1-score, FPS, GFLOPs, and memory usage under different input resolutions.

**Response to Major Comment 4:**

Thank you for this constructive suggestion. We agree that input resolution affects detection accuracy, inference speed, and memory usage, and therefore a systematic analysis of input scale is necessary. Following the reviewer’s suggestion, we added an input-resolution ablation study in the revised manuscript. Specifically, four input sizes, including 1024 × 1024, 1280 × 1280, 1536 × 1536, and 2048 × 2048, were compared while keeping the model architecture, training strategy, and evaluation protocol unchanged.

The results show that Recall and F1 generally improved as the input resolution increased. F1 increased from 92.4% at 1024 × 1024 to 95.8% at 2048 × 2048, and Recall increased from 88.2% to 92.7%, indicating that higher-resolution inputs help preserve the edges, strokes, and weak texture details of small engraved codes. Meanwhile, the computation increased from 60.13 GFLOPs to 240.54 GFLOPs, GPU FPS decreased from 128.1 to 33.6, CPU FPS decreased from 2.92 to 1.06, and peak memory increased from 298.58 MB to 1096.51 MB. These results indicate that higher input resolution introduces greater computational and memory overhead. Since this study primarily focuses on accurate detection of small engraved codes under challenging imaging conditions, 2048 × 2048 was adopted as the default input resolution for the self-collected dataset.

**Major Comment 5:**

Deployment experiments on realistic platforms should be added. The manuscript emphasizes lightweight design and practical application. However, the current speed evaluation appears to be mainly conducted on a high-performance GPU platform. For a practical surgical instrument code detection system, FPS on a high-end GPU alone is not sufficient to demonstrate real-world deployability. The authors are encouraged to evaluate the proposed model on more realistic platforms, such as CPU, Jetson devices, embedded GPUs, or ordinary industrial workstations. In addition to FPS, memory consumption, model size, and end-to-end latency should also be reported. This would better support the claimed practical value of the proposed method.

**Response to Major Comment 5:**

Thank you for this constructive suggestion. We agree that reporting speed results only on a high-performance GPU is insufficient to fully support the practical deployment value of the proposed model. Following the reviewer’s comment, we added a more complete evaluation of efficiency and resource consumption in the revised manuscript. On the self-collected surgical instrument code dataset, we reported the number of parameters, computational cost, GPU FPS, CPU FPS, and peak GPU memory of different models. All FPS values were measured on the same workstation platform with a batch size of 1, and the timing included preprocessing, model inference, and post-processing rather than only the network forward time. Therefore, the corresponding average end-to-end processing latency can be directly derived from the measured FPS.

The additional results show that LA-DBNet achieves the highest F1 while maintaining relatively low model complexity and resource consumption. Compared with DBNet, LA-DBNet improves the F1 from 92.2% to 95.8%, reduces the number of parameters from 25.41 M to 3.35 M, and decreases the computational cost from 471.47 GFLOPs to 240.54 GFLOPs. Meanwhile, GPU FPS increases from 18.5 to 33.6, CPU FPS increases from 0.75 to 1.06, and peak GPU memory decreases from 1794.62 MB to 1096.51 MB. Based on the measured end-to-end FPS, the average processing latency of LA-DBNet is approximately 29.8 ms/image on GPU and 943 ms/image on CPU, corresponding to the measured GPU FPS of 33.6 and CPU FPS of 1.06, respectively. These results indicate that the proposed method achieves a favorable balance among detection accuracy, model size, inference speed, and resource usage.

We also agree with the reviewer that evaluation on Jetson devices, embedded GPUs, or industrial edge-computing platforms would further strengthen the practical validation. Due to the limitation of currently available experimental platforms, we were unable to conduct additional tests on these platforms at this stage. Therefore, in the revised manuscript, we avoided presenting the current results as a complete embedded deployment validation. Instead, we describe them as a preliminary deployment-feasibility evaluation under GPU and CPU-only settings. We have also added a statement in the Discussion section that future work will further evaluate and optimize the proposed model on Jetson devices, embedded GPUs, or industrial edge-computing platforms to more systematically validate its application value in practical surgical instrument traceability systems.

**Major Comment 6:**

Comparisons with more recent text detection methods should be included. It is suggested that the authors investigate whether there are any recently proposed models with open-source implementations. If available, some of these newer models should be included for comparison to improve the persuasiveness of the experimental results.

**Response to Major Comment 6:**

Thank you for this constructive suggestion. We agree that including more recent detection methods with publicly available implementations can further improve the completeness and persuasiveness of the comparative experiments. Following the reviewer’s suggestion, we revisited recent reproducible text detection/OCR detection frameworks and real-time object detection methods, and added PP-OCRv4 and YOLOv11 as representative comparison methods in the revised manuscript.

Specifically, PP-OCRv4 is a widely used open-source OCR system with a mature engineering implementation, and its detection branch can serve as a representative text-region localization method in practical OCR pipelines. YOLOv11 is a recent real-time detector from the YOLO family, providing a favorable balance between accuracy and inference efficiency, and was included as a representative general real-time detection framework for the surgical instrument code detection task.

All newly added methods were evaluated under the same data split, input resolution, evaluation metrics, and hardware platform to ensure fairness and reproducibility. The revised experimental results show that LA-DBNet achieves favorable overall detection performance on the self-collected surgical instrument code dataset while maintaining a compact model size and good inference efficiency. The corresponding descriptions and additional experimental results have been added to the comparative experiment section of the revised manuscript.

**Major Comment 8:**

Visualization results and failure-case analysis are insufficient. The current visualization results are relatively limited. More qualitative results should be added, especially under challenging conditions such as severe specular reflection, motion blur, low contrast, worn engravings, curved metallic surfaces, occlusion, and small-scale code regions. In addition, failure-case analysis should be provided. The authors should discuss under what conditions the proposed method still fails and explain the possible reasons. This is important for clarifying the limitations and application boundaries of the method. Since the proposed DECA module is designed to enhance edge and alignment features, the authors are also encouraged to provide visual analyses related to this module, such as edge-response maps, attention maps, or feature activation maps, to more intuitively demonstrate its effectiveness for weak-boundary code detection.

**Response to Major Comment 8:**

Thank you for the suggestion. Following the reviewer’s comment, we added visualization and qualitative analyses in the revised manuscript, including DECA feature-response heatmaps, detection results under challenging degraded conditions, and typical failure cases. The DECA visualization shows that the discrepancy map and dual-gate responses are relatively activated around code regions and neighboring boundaries, further indicating that the module helps enhance cross-scale boundary responses and local structural representation. The qualitative results show that LA-DBNet can still localize code regions under reflection, blur, wear, weak-boundary, and low-contrast conditions. We also added failure-case analysis, showing that false positives, missed detections, or localization shifts may still occur under strong reflection, text-like structural edges, and extremely weak engraving. The corresponding content has been added to the visualization and qualitative analysis section of the revised manuscript.

**Major Comment 9:**

Data and code availability should be strengthened. The core experiments of this manuscript rely on a self-collected surgical instrument code dataset. To ensure reproducibility, the authors should provide a public data repository, DOI, or supplementary dataset archive. Ideally, the complete images, annotation files, training/validation/testing splits, difficult-subset definition, source code, and training configuration files should be made available. If the dataset cannot be fully released due to privacy, institutional, or other restrictions, the authors should clearly explain these limitations and provide as much information as possible, such as anonymized examples, annotation format, dataset statistics, and reproducible experimental configurations. Otherwise, the reproducibility and credibility of the experimental conclusions may be weakened.

**Response to Major Comment 9:**

Thank you for this important suggestion. We agree that data and code availability is essential for improving the reproducibility and credibility of the experimental results. Following the reviewer’s suggestion, we have organized and uploaded the source code and related experimental resources to a public GitHub repository:

<https://github.com/wy49497/LADBNet>

The repository includes the implementation of LA-DBNet, training scripts, testing scripts, main configuration files, trained model weights, annotation examples, and the complete self-collected surgical instrument code dataset used in this study, including the training, validation, and test sets. The corresponding annotation files and dataset split information are also provided. These resources allow readers to reproduce the main experimental results and understand the data format, annotation protocol, and experimental configuration used in this study.

**Minor Comment 1:**

The notation of evaluation metrics should be consistent throughout the manuscript. For example, if the F1-score is used as the main metric, the notation should be consistently written as “F1-score” or “F1” rather than using different symbols in different sections.

**Response to Minor Comment 1:**

Thank you for this careful comment. We have checked and revised the notation of evaluation metrics throughout the manuscript. Precision, Recall, and F1 score are first defined as P, R, and F1, respectively, and the abbreviated forms are used consistently thereafter. The table headers and descriptions in the main text have also been unified accordingly.

**Minor Comment 2:**

All result tables should clearly indicate the corresponding dataset, input resolution, and evaluation setting in the table caption or footnote. For example, captions such as “Results on the self-collected surgical instrument code dataset at 2048 × 2048 input resolution” or “Results on ICDAR2015 at 1024 × 1024 input resolution” would help avoid confusion.

**Response to Minor Comment 2:**

Thank you for this helpful suggestion. We have revised the captions to specify the corresponding dataset, input resolution, and evaluation setting. For experiments on the self-collected surgical instrument code dataset, we indicate that the input resolution is 2048 × 2048. For experiments on ICDAR2015, we indicate that the input resolution is 1024 × 1024. We also clarified the hardware and batch-size settings used for speed evaluation where applicable.
