# Supplementary material for: A lightweight alignment-aware DBNet for surgical instrument code detection
Source: PLoS One. 2026 Aug 7;21(8):e0355611. doi: 10.1371/journal.pone.0355611 (PMC13450737; doi:10.1371/journal.pone.0355611)
Supplement: Supplementary file 3 [file pone.0355611.s004.docx]

Response to Reviewers

Manuscript number: PONE-D-26-18770R1

Title: A lightweight alignment-aware DBNet for surgical instrument code detection

Dear Editor and Reviewers,

We sincerely thank you for your careful evaluation of our manuscript and for your thoughtful and constructive comments. We greatly appreciate the time and effort you have devoted to reviewing our work. In response to the comments and suggestions provided, we have carefully re-examined the entire manuscript and revised the relevant sections in accordance with the journal’s requirements. We have also conducted a thorough check of the manuscript to improve its clarity, accuracy, transparency, and overall presentation. All changes have been clearly marked in the revised manuscript, and our detailed point-by-point responses are provided below.

**Editor Comment 1:**

Data and code availability: Please ensure that the Data Availability Statement complies with PLOS ONE policy. Clarify whether the dataset, annotations, trained model weights, and/or source code are publicly available. If any of these cannot be shared, please provide an appropriate justification.

**Response to Comment 1:**

Thank you for this important comment. We have revised the Data Availability Statement to clearly describe the availability of all research materials associated with this study. The datasets, annotation files, trained model weights, and source code used in this work are publicly available without restriction through our GitHub repository at: <https://github.com/wy49497/LADBNet>.

**Editor Comment 2:**

Statistical robustness: Please clarify whether the reported results are based on a single training run or multiple independent runs. If only a single run was performed, please acknowledge this as a limitation.

**Response to Comment 2:**

Thank you for raising this important point. We have clarified in the revised manuscript that the reported results were obtained from a single independent training and evaluation run for each model. Therefore, the reported values represent the performance observed in that individual run rather than the mean performance across multiple runs with different random seeds.

We acknowledge that a single run does not capture the potential variability arising from random initialization, stochastic optimization, and data sampling. We have therefore explicitly added this issue as a limitation in the Discussion section. In future work, we will conduct multiple independent experiments using different random seeds and report the results as the mean and standard deviation, or with appropriate confidence intervals, to provide a more comprehensive assessment of statistical robustness.

**Editor Comment 3:**

Generalizability: Please moderate statements suggesting broad generalizability, as the proposed method has been validated primarily on a single proprietary surgical dataset and one public benchmark.

**Response to Comment 3:**

Thank you very much for this valuable comment. We agree that the generalizability of the proposed method should be interpreted with caution, since the current experimental evaluation was conducted primarily on one self-collected surgical instrument dataset and one public benchmark dataset. Accordingly, we have carefully reviewed the entire manuscript and appropriately moderated the relevant statements, particularly in the Datasets section and Conclusion, to avoid implying broad or universal generalizability.

**Editor Comment 4:**

Evaluation protocol: Please provide a brief clarification that all comparative methods were evaluated under identical experimental conditions and specify any factors that may affect the reported inference speed (e.g., framework, implementation, and hardware).

**Response to Comment 4:**

Thank you very much for this important comment. We have clarified the evaluation protocol in the revised manuscript. All experiments conducted within each dataset adopted identical data splits, input resolutions, evaluation metrics, test hardware platforms, and evaluation protocols. For methods with publicly available implementations, their official training and post-processing settings were retained as much as possible.

We have also clarified that the reported inference speed was measured under the experimental environment listed in Table 1 and may be affected by factors such as the deep-learning framework, code implementation and optimization strategy, hardware configuration, batch size, and input resolution.

We sincerely appreciate the Academic Editor’s constructive comments, which have helped us further improve the clarity, transparency, and rigor of the manuscript.

Sincerely,

The Authors
