# Supplementary material for: A lightweight alignment-aware DBNet for surgical instrument code detection
Source: PLoS One. 2026 Aug 7;21(8):e0355611. doi: 10.1371/journal.pone.0355611 (PMC13450737; doi:10.1371/journal.pone.0355611)
Supplement: Supplementary file 4 [file pone.0355611.s005.docx]

Response to Reviewers

Manuscript number: PONE-D-26-18770R2

Title: A lightweight alignment-aware DBNet for surgical instrument code detection

Dear Editor,

Thank you for your careful evaluation of our revised manuscript and for bringing this omission to our attention. We sincerely apologise that the Data Availability Statement was inadvertently omitted from the previous revised manuscript file, although the relevant information had been entered in the submission system.

In response to your comment, we have now added a separate “Data Availability Statement” directly before the References section of the revised manuscript.

We have also verified that the research materials are publicly accessible. The image dataset has been deposited in Zenodo and is publicly available at <https://doi.org/10.5281/zenodo.21452216>. The annotation files, trained model weights, source code, and corresponding usage instructions are publicly available in the GitHub repository at [https://github.com/wy49497/LADBNet](https://github.com/wy49497/LADBNet?utm_source=chatgpt.com).

**Editor Comment 1:**

Data Availability Statement (Comment 1): Your response states that the Data Availability Statement has been revised to reference your GitHub repository, but I was unable to locate this statement anywhere in the manuscript file. Please add the Data Availability Statement directly to the manuscript (not only the submission system field), and confirm that the repository is public and contains the dataset, annotations, trained model weights, and source code as described.

**Response to Comment 1:**

Thank you for pointing this out. We sincerely apologise for the omission of the Data Availability Statement from the previous revised manuscript file. Although the relevant information had been provided in the submission system, the statement was inadvertently omitted from the manuscript itself.

We have now added a separate “Data Availability Statement” directly before the References section of the revised manuscript. We confirm that the image dataset has been deposited in Zenodo and is publicly accessible at <https://doi.org/10.5281/zenodo.21452216>. The annotation files, trained model weights, source code, and corresponding usage instructions are publicly accessible through our GitHub repository at [https://github.com/wy49497/LADBNet](https://github.com/wy49497/LADBNet?utm_source=chatgpt.com).

The following statement has been added to the manuscript:

“Data Availability Statement: The image dataset underlying the findings of this study is publicly available in the Zenodo repository at <https://doi.org/10.5281/zenodo.21452216>. The annotation files, trained model weights, source code, and instructions for accessing and using these resources are publicly available in the GitHub repository at https://github.com/wy49497/LADBNet.”

Thank you for bringing this omission to our attention. We have uploaded the corrected manuscript accordingly.

Sincerely,

The Authors
