# Supplementary material for: A lightweight alignment-aware DBNet for surgical instrument code detection
Source: PLoS One. 2026 Aug 7;21(8):e0355611. doi: 10.1371/journal.pone.0355611 (PMC13450737; doi:10.1371/journal.pone.0355611)
Supplement: Peer review history

# Peer review: Decision Letter 0

**DOI:** 10.1371/journal.pone.0355611.r001

**Authors:** Athanasios Pantelis

9 Jun 2026

PONE-D-26-18770

A lightweight alignment-aware DBNet for surgical instrument code detection

PLOS One

Dear Dr.  Qu,

Thank you for submitting your manuscript to PLOS ONE. After careful consideration, we feel that it has merit but does not fully meet PLOS ONE’s publication criteria as it currently stands. Therefore, we invite you to submit a revised version of the manuscript that addresses the points raised during the review process.

Please submit your revised manuscript by Jul 24 2026 11:59PM. If you will need more time than this to complete your revisions, please reply to this message or contact the journal office at plosone@plos.org. When you're ready to submit your revision, log on to https://www.editorialmanager.com/pone/ and select the 'Submissions Needing Revision' folder to locate your manuscript file.

Please include the following items when submitting your revised manuscript:

- A letter that responds to each point raised by the academic editor and reviewer(s). You should upload this letter as a separate file labeled 'Response to Reviewers'.
- A marked-up copy of your manuscript that highlights changes made to the original version. You should upload this as a separate file labeled 'Revised Manuscript with Track Changes'.
- An unmarked version of your revised paper without tracked changes. You should upload this as a separate file labeled 'Manuscript'.

If you would like to make changes to your financial disclosure, please include your updated statement in your cover letter. Guidelines for resubmitting your figure files are available below the reviewer comments at the end of this letter.

If applicable, we recommend that you deposit your laboratory protocols in protocols.io to enhance the reproducibility of your results. Protocols.io assigns your protocol its own identifier (DOI) so that it can be cited independently in the future. For instructions see: https://journals.plos.org/plosone/s/submission-guidelines#loc-laboratory-protocols. Additionally, PLOS ONE offers an option for publishing peer-reviewed Lab Protocol articles, which describe protocols hosted on protocols.io. Read more information on sharing protocols at https://plos.org/protocols?utm_medium=editorial-email&utm_source=authorletters&utm_campaign=protocols.

As the corresponding author, your ORCID iD is verified in the submission system and will appear in the published article. PLOS supports the use of ORCID, and we encourage all coauthors to register for an ORCID iD and use it as well. Please encourage your coauthors to verify their ORCID iD within the submission system before final acceptance, as unverified ORCID iDs will not appear in the published article. *Only*  the individual author can complete the verification step; PLOS staff *cannot*  verify ORCID iDs on behalf of authors.

We look forward to receiving your revised manuscript.

Kind regards,

Athanasios G. Pantelis

Academic Editor

PLOS One

Journal Requirements:

When submitting your revision, we need you to address these additional requirements.

1.Please ensure that your manuscript meets PLOS ONE's style requirements, including those for file naming. The PLOS ONE style templates can be found at

https://journals.plos.org/plosone/s/file?id=wjVg/PLOSOne_formatting_sample_main_body.pdf and

https://journals.plos.org/plosone/s/file?id=ba62/PLOSOne_formatting_sample_title_authors_affiliations.pdf

2. Please note that PLOS One has specific guidelines on code sharing for submissions in which author-generated code underpins the findings in the manuscript. In these cases, we expect all author-generated code to be made available without restrictions upon publication of the work. Please review our guidelines at https://journals.plos.org/plosone/s/materials-and-software-sharing#loc-sharing-code and ensure that your code is shared in a way that follows best practice and facilitates reproducibility and reuse.

3. We note that your Data Availability Statement is currently as follows: All relevant data are within the manuscript and in Supporting Information files.

Please confirm at this time whether or not your submission contains all raw data required to replicate the results of your study. Authors must share the “minimal data set” for their submission. PLOS defines the minimal data set to consist of the data required to replicate all study findings reported in the article, as well as related metadata and methods (https://journals.plos.org/plosone/s/data-availability#loc-minimal-data-set-definition).

For example, authors should submit the following data:

- The values behind the means, standard deviations and other measures reported;

- The values used to build graphs;

- The points extracted from images for analysis.

Authors do not need to submit their entire data set if only a portion of the data was used in the reported study.

If your submission does not contain these data, please either upload them as Supporting Information files or deposit them to a stable, public repository and provide us with the relevant URLs, DOIs, or accession numbers. For a list of recommended repositories, please see https://journals.plos.org/plosone/s/recommended-repositories.

If there are ethical or legal restrictions on sharing a de-identified data set, please explain them in detail (e.g., data contain potentially sensitive information, data are owned by a third-party organization, etc.) and who has imposed them (e.g., an ethics committee). Please also provide contact information for a data access committee, ethics committee, or other institutional body to which data requests may be sent. If data are owned by a third party, please indicate how others may request data access.

4. If the reviewer comments include a recommendation to cite specific previously published works, please review and evaluate these publications to determine whether they are relevant and should be cited. There is no requirement to cite these works unless the editor has indicated otherwise.

[Note: HTML markup is below. Please do not edit.]

Reviewers' comments:

Reviewer's Responses to Questions

**Comments to the Author**

1. Is the manuscript technically sound, and do the data support the conclusions?

The manuscript must describe a technically sound piece of scientific research with data that supports the conclusions. Experiments must have been conducted rigorously, with appropriate controls, replication, and sample sizes. The conclusions must be drawn appropriately based on the data presented.

Reviewer #1: No

Reviewer #2: Yes

**********

2. Has the statistical analysis been performed appropriately and rigorously?

Reviewer #1: No

Reviewer #2: Yes

**********

3. Have the authors made all data underlying the findings in their manuscript fully available?

The PLOS Data policy requires authors to make all data underlying the findings described in their manuscript fully available without restriction, with rare exception (please refer to the Data Availability Statement in the manuscript PDF file). The data should be provided as part of the manuscript or its supporting information, or deposited to a public repository. For example, in addition to summary statistics, the data points behind means, medians and variance measures should be available. If there are restrictions on publicly sharing data—e.g. participant privacy or use of data from a third party—those must be specified.

Reviewer #1: No

Reviewer #2: No

**********

4. Is the manuscript presented in an intelligible fashion and written in standard English?

PLOS ONE does not copyedit accepted manuscripts, so the language in submitted articles must be clear, correct, and unambiguous. Any typographical or grammatical errors should be corrected at revision, so please note any specific errors here.

Reviewer #1: Yes

Reviewer #2: Yes

**********

5. Review Comments to the Author

Please use the space provided to explain your answers to the questions above. You may also include additional comments for the author, including concerns about dual publication, research ethics, or publication ethics. (Please upload your review as an attachment if it exceeds 20,000 characters)

Reviewer #1: This manuscript addresses industry challenges in detecting engraved codes on surgical instrument surfaces, such as metal reflections, motion blur, scale variations, and weak textures, by proposing a lightweight alignment-aware DBNet model (LA-DBNet). Based on the DBNet framework, the model employs MobileNetV4 combined with LiteFPN to construct a lightweight feature extraction and fusion network. It incorporates a directional edge-cooperative alignment (DECA) module to improve the spatial alignment of cross-scale features. It embeds the ECA attention mechanism in the high-resolution feature layer to enhance target responsiveness and introduces a Region-Weighted Consistency Learning (RWCL) strategy to improve detection robustness for degraded samples. However, the manuscript requires certain modifications and improvements. My comments are as follows:

(1) The manuscript mentions that only the highest-resolution output feature P2 from LiteFPN is retained as input to the detection head, yet it simultaneously inserts the DECA module into LiteFPN’s complete top-down fusion path for cross-scale alignment. If only P2 features are ultimately used, why is it necessary to construct a complete four-layer feature pyramid and perform DECA fusion across all adjacent scales? It is recommended to explicitly state which features are ultimately used for detection and to clarify the actual role of cross-scale fusion.

(2) Directional enhancement relies solely on deep convolutions in two orthogonal directions (1×5 and 5×1), which fails to cover the oblique characters commonly found in surgical instrument codes. The manuscript does not explain why multi-directional convolutions or rotation-invariant feature extraction were not adopted, nor does it compare the performance differences among various directional combinations.

(3) The difference map Δ directly uses the absolute difference of feature values. This operation can only characterize pixel-level intensity differences and cannot effectively capture spatial misalignment or semantic differences between cross-scale features. The manuscript does not demonstrate that the absolute difference is the optimal difference metric, nor does it compare the performance of other metrics such as cosine similarity or Euclidean distance.

(4) Why does the manuscript require two independent spatial gates to weight shallow and deep features separately? Why not adopt a simpler single-gate mechanism to control the fusion ratio between the two? There is a lack of ablation experiments to validate the necessity of the dual-gate design.

(5) The foreground mask in the manuscript is generated based on the probability map of the clean branch, and the threshold τ = 0.2 is an empirical value. The authors did not conduct a hyperparameter sensitivity analysis, so they cannot prove the universality of this threshold. Errors in the predictions of some clean branches can lead to an inaccurate foreground mask, which in turn causes the consistency loss to generate erroneous supervision signals.

(6) The manuscript does not compare the proposed method with the latest lightweight text detection models, such as DBNet++, the MobileNetV3 backbone, YOLOv8/YOLOv9-based models, or the detection components of PP-OCRv3/v4. These models may offer advantages in terms of accuracy and speed.

(7) The manuscript’s ablation experiments only verify the overall contributions of major modules and do not perform fine-grained ablation on key components within the modules. For example, what are the individual contributions of the direction enhancement, difference prompt, and dual-gating components within the DECA module? How much would removing any one of these components affect performance?

(8) The manuscript mentions that the dataset is split at the instance level, but does not specify the exact splitting criteria or process. For example, is the split based on instrument instances or categories? How many images are there per instance? Does the splitting ensure category balance between the training and test sets?

(9) The manuscript states that the default training and post-processing settings from the official implementation were retained, but no model-specific tuning was performed for the surgical instrument code dataset. Since LA-DBNet was specifically designed for this task, is this comparison method fair to other models?

(10) The manuscript only implements surgical instrument code detection and does not include a character recognition module, whereas an actual traceability system requires a complete detection and recognition pipeline. The authors do not address the compatibility of LA-DBNet with existing recognition models nor provide end-to-end recognition accuracy results, making it impossible to assess its practical application value.

Reviewer #2: This manuscript focuses on surgical instrument code detection and proposes a lightweight alignment-aware DBNet-based method. The study aims to address the difficulty of detecting engraved or printed codes on metallic surgical instruments under challenging imaging conditions, such as specular reflection, motion blur, low contrast, and weak object boundaries. Based on the DBNet framework, the authors introduce a lightweight backbone, a multi-scale feature fusion module, a directional edge collaborative alignment module, and a region-weighted consistency learning strategy.

Overall, the topic has practical significance, especially for surgical instrument traceability, sterilization management, and medical safety. The proposed framework is relatively complete, and the reported results show certain improvements over the baseline model on the self-collected dataset.

However, the current manuscript still has several important issues that need to be addressed. In particular, the justification of key hyperparameters is insufficient, the input-resolution settings are not clearly explained, the FPS evaluation protocol is ambiguous, the fairness of experimental comparisons needs further clarification, comparisons with recent methods are inadequate, and the reproducibility of the self-collected dataset remains unclear. Therefore, I recommend major revision before the manuscript can be considered for publication.

Major Comments:

1. Insufficient justification of key hyperparameters.

The proposed region-weighted consistency learning strategy involves several important hyperparameters, such as τ, α, and β. These parameters directly affect foreground-region selection, consistency-loss weighting, and training stability. However, the manuscript does not sufficiently explain how these parameters were determined.

The authors should clarify whether these values were selected based on validation-set tuning, empirical experience, or prior literature. A sensitivity analysis should also be provided to show how the model performance changes under different settings of τ, α, and β. In particular, τ appears to be an important threshold for foreground-region selection, and inappropriate selection may cause the model to focus on unreliable prediction regions or even introduce self-reinforcing errors.

2. The resizing strategy for ICDAR2015 needs clarification.

The manuscript states that, due to substantial differences between the self-collected surgical instrument code dataset and ICDAR2015 in terms of original image resolution, target-scale distribution, and scene characteristics, the input images were resized to 2048 × 2048 and 1024 × 1024, respectively.

However, ICDAR2015 images usually have a non-square aspect ratio close to 1280 × 720. If these images are directly resized to 1024 × 1024, the original aspect ratio will be changed, which may distort the geometry, scale distribution, and shape of text instances.

Therefore, the authors should clearly specify the resizing strategy used for ICDAR2015. Was direct stretching to 1024 × 1024 applied, or was aspect-ratio-preserving resizing followed by padding used? If direct resizing was used, the authors should discuss its potential influence on text geometry and detection results. If padding was used, this should be explicitly stated in the experimental settings. The rationale for choosing 1024 × 1024 should also be provided.

3. The FPS evaluation protocol and result source should be clearly specified.

The manuscript reports FPS values, but the testing conditions are not sufficiently clear. Since the self-collected dataset and ICDAR2015 use different input resolutions, for example 2048 × 2048 and 1024 × 1024, the inference speed may vary substantially. Therefore, each FPS result should be clearly associated with its corresponding dataset, input resolution, and testing protocol.

4. An input-resolution ablation study is recommended.

The current experiments mainly use relatively high input resolutions. High-resolution input can preserve more fine-grained texture and boundary information of surgical instrument codes, which may improve detection performance. However, for a lightweight detection model, reducing the input resolution is also an important way to improve inference efficiency, reduce computational cost, and enhance deployability.

I suggest that the authors add an input-resolution ablation study, for example by comparing 2048 × 2048, 1536 × 1536, 1280 × 1280, and 1024 × 1024. The authors should report Precision, Recall, F1-score, FPS, GFLOPs, and memory usage under different input resolutions.

5. Deployment experiments on realistic platforms should be added.

The manuscript emphasizes lightweight design and practical application. However, the current speed evaluation appears to be mainly conducted on a high-performance GPU platform. For a practical surgical instrument code detection system, FPS on a high-end GPU alone is not sufficient to demonstrate real-world deployability.

The authors are encouraged to evaluate the proposed model on more realistic platforms, such as CPU, Jetson devices, embedded GPUs, or ordinary industrial workstations. In addition to FPS, memory consumption, model size, and end-to-end latency should also be reported. This would better support the claimed practical value of the proposed method.

6. Comparisons with more recent text detection methods should be included.

It is suggested that the authors investigate whether there are any recently proposed models with open-source implementations. If available, some of these newer models should be included for comparison to improve the persuasiveness of the experimental results.

8. Visualization results and failure-case analysis are insufficient.

The current visualization results are relatively limited. More qualitative results should be added, especially under challenging conditions such as severe specular reflection, motion blur, low contrast, worn engravings, curved metallic surfaces, occlusion, and small-scale code regions.

In addition, failure-case analysis should be provided. The authors should discuss under what conditions the proposed method still fails and explain the possible reasons. This is important for clarifying the limitations and application boundaries of the method.

Since the proposed DECA module is designed to enhance edge and alignment features, the authors are also encouraged to provide visual analyses related to this module, such as edge-response maps, attention maps, or feature activation maps, to more intuitively demonstrate its effectiveness for weak-boundary code detection.

9. Data and code availability should be strengthened.

The core experiments of this manuscript rely on a self-collected surgical instrument code dataset. To ensure reproducibility, the authors should provide a public data repository, DOI, or supplementary dataset archive. Ideally, the complete images, annotation files, training/validation/testing splits, difficult-subset definition, source code, and training configuration files should be made available.

If the dataset cannot be fully released due to privacy, institutional, or other restrictions, the authors should clearly explain these limitations and provide as much information as possible, such as anonymized examples, annotation format, dataset statistics, and reproducible experimental configurations. Otherwise, the reproducibility and credibility of the experimental conclusions may be weakened.

Minor Comments

1. The notation of evaluation metrics should be consistent throughout the manuscript. For example, if the F1-score is used as the main metric, the notation should be consistently written as “F1-score” or “F1” rather than using different symbols in different sections.

2. All result tables should clearly indicate the corresponding dataset, input resolution, and evaluation setting in the table caption or footnote. For example, captions such as “Results on the self-collected surgical instrument code dataset at 2048 × 2048 input resolution” or “Results on ICDAR2015 at 1024 × 1024 input resolution” would help avoid confusion.

**********

6. PLOS authors have the option to publish the peer review history of their article (what does this mean?). If published, this will include your full peer review and any attached files.

If you choose “no”, your identity will remain anonymous but your review may still be made public.

**Do you want your identity to be public for this peer review?**  For information about this choice, including consent withdrawal, please see our Privacy Policy.

Reviewer #1: No

Reviewer #2: No

**********

[NOTE: If reviewer comments were submitted as an attachment file, they will be attached to this email and accessible via the submission site. Please log into your account, locate the manuscript record, and check for the action link "View Attachments". If this link does not appear, there are no attachment files.]

To ensure your figures meet our technical requirements, please review our figure guidelines: https://journals.plos.org/plosone/s/figures

You may also use PLOS’s free figure tool, NAAS, to help you prepare publication quality figures: https://journals.plos.org/plosone/s/figures#loc-tools-for-figure-preparation.

NAAS will assess whether your figures meet our technical requirements by comparing each figure against our figure specifications.

Attachment Submitted filename: Comments.pdf

# Author comment: Author response to Decision Letter 1

**DOI:** 10.1371/journal.pone.0355611.r002

27 Jun 2026

Manuscript number: PONE-D-26-18770

Title: A lightweight alignment-aware DBNet for surgical instrument code detection

Dear Editor and Reviewers,

We sincerely thank the editor and the two reviewers for their careful evaluation of our manuscript entitled “A lightweight alignment-aware DBNet for surgical instrument code detection” and for their valuable comments and suggestions. These comments have been very helpful in improving the experimental design, methodological description, and result analysis of our work. Following these suggestions, we have thoroughly revised the manuscript by adding additional experiments, clarifying the method description, and improving the discussion of the results. In this response letter, we provide point-by-point responses to the reviewers’ comments and describe the corresponding revisions made in the manuscript. We sincerely appreciate the constructive feedback from the editor and reviewers.

Our detailed responses are provided below.

Reviewer #1

Comment 1:

The manuscript mentions that only the highest-resolution output feature P2 from LiteFPN is retained as input to the detection head, yet it simultaneously inserts the DECA module into LiteFPN’s complete top-down fusion path for cross-scale alignment. If only P2 features are ultimately used, why is it necessary to construct a complete four-layer feature pyramid and perform DECA fusion across all adjacent scales? It is recommended to explicitly state which features are ultimately used for detection and to clarify the actual role of cross-scale fusion.

Response to Comment 1:

Thank you for this important comment. We agree that the relationship between the complete LiteFPN pathway and the final P2 feature should be clarified. In the revised manuscript, we have explicitly stated that only the highest-resolution output feature P2 is fed into the detection head. However, this P2 feature is not a purely shallow feature. It is generated after the complete top-down LiteFPN fusion process and therefore already incorporates semantic information propagated from deeper feature levels. The purpose of constructing the four-level feature pyramid is to allow high-level semantic cues to be progressively transferred to the high-resolution feature map, while the DECA modules improve cross-scale alignment between adjacent feature levels during this process. Therefore, although only P2 is used for final prediction, the complete feature pyramid and DECA-based fusion are still necessary for generating a semantically enriched and structurally aligned high-resolution feature map for detection.

Comment 2:

Directional enhancement relies solely on deep convolutions in two orthogonal directions (1×5 and 5×1), which fails to cover the oblique characters commonly found in surgical instrument codes. The manuscript does not explain why multi-directional convolutions or rotation-invariant feature extraction were not adopted, nor does it compare the performance differences among various directional combinations.

Response to Comment 2:

Thank you for this valuable suggestion. We agree that simply stating the use of two orthogonal directional convolution branches, i.e., 1×5 and 5×1, is not sufficient. Therefore, in the revised manuscript, we added a fine-grained ablation study on the directional enhancement component of DECA to compare the effects of different directional modeling strategies.

Specifically, we compared several configurations, including removing Directional Enhancement, replacing directional convolution with 3×3 DWConv, using only the 5×1 branch, using only the 1×5 branch, and adding an extra 3×3 branch to the 1×5 and 5×1 branches. The results show that removing Directional Enhancement reduced F1 from 93.8% to 91.4%, and Recall from 90.0% to 86.4%, indicating that directional enhancement plays an important role in reducing missed detections. Using only the 5×1 or 1×5 branch achieved F1 values of 92.3% and 93.0%, respectively. Replacing directional convolution with 3×3 DWConv resulted in an F1 of 92.5%. Adding an additional 3×3 branch to the 1×5 and 5×1 branches achieved an F1 of 93.4%, which was still lower than that of Full DECA, i.e., 93.8%.

These results indicate that the two orthogonal 1×5 and 5×1 branches can effectively model elongated strokes and edge continuity in engraved surgical instrument codes. Although this design is not an explicit rotation-invariant feature extraction method and cannot fully cover all oblique character patterns, it achieved better overall performance in this task than single-direction branches, standard 3×3 convolution, or the additional 3×3-branch configuration. Considering the lightweight design and inference efficiency requirements of the proposed model, we retained the 1×5 + 5×1 dual-direction design as the directional enhancement module. The corresponding results and analysis have been added to the fine-grained DECA ablation section of the revised manuscript.

Comment 3:

The difference map Δ directly uses the absolute difference of feature values. This operation can only characterize pixel-level intensity differences and cannot effectively capture spatial misalignment or semantic differences between cross-scale features. The manuscript does not demonstrate that the absolute difference is the optimal difference metric, nor does it compare the performance of other metrics such as cosine similarity or Euclidean distance.

Response to Comment 3:

Thank you for this insightful comment. We agree that the role and limitation of the absolute-difference operation should be more clearly explained. In the revised manuscript, we have clarified that the discrepancy map Δ is not computed from raw image pixel intensities. Instead, it is calculated from the direction-enhanced shallow feature and the direction-enhanced upsampled high-level feature after they have been projected to the same spatial scale and channel space. Therefore, Δ represents a local feature-response discrepancy rather than a raw pixel-intensity difference.

After convolutional encoding and directional enhancement, the feature responses contain information related to code-region edges, strokes, local textures, and semantic discrimination. Thus, the absolute-difference operation is used as a lightweight cue to highlight local response inconsistencies between shallow detail features and high-level semantic features, especially around weak boundaries and discontinuous engraved strokes. We have also clarified that this operation is not intended to serve as an explicit geometric registration module for estimating large spatial displacement. Instead, it provides a local discrepancy prompt, which is further processed together with the direction-enhanced features through an edge projection mapping. This enables the network to learn context-aware structural cues related to boundary misalignment, weak edges, and cross-scale response inconsistency.

To further justify the choice of the discrepancy metric, we added a fine-grained ablation experiment in the revised manuscript. Specifically, we replaced the absolute-difference operation with a cosine-distance-based alternative. The results show that the full DECA using absolute difference achieves an F1 score of 93.8%, whereas replacing it with cosine distance decreases the F1 score to 93.4%. This indicates that, under the present experimental setting, the position-wise absolute difference is slightly more effective for preserving response-magnitude differences around local boundaries and weak-texture regions. In contrast, cosine distance mainly focuses on feature-direction similarity and may weaken amplitude information related to edge-strength variation and local structural discontinuity.

We have revised the manuscript accordingly. We also avoided claiming that absolute difference is universally optimal. Instead, we state that it provides a favorable balance between detection performance and computational cost for the present surgical instrument code detection task.

Comment 4:

Why does the manuscript require two independent spatial gates to weight shallow and deep features separately? Why not adopt a simpler single-gate mechanism to control the fusion ratio between the two? There is a lack of ablation experiments to validate the necessity of the dual-gate design.

Response to Comment 4:

Thank you for this helpful suggestion. In the revised manuscript, we added a fine-grained ablation experiment by replacing the dual-gate design in DECA with a single-gate mechanism. The results show that the full DECA achieves an F1 score of 93.8%, whereas the single-gate variant decreases the F1 score to 92.8%. This confirms that independently recalibrating shallow detail features and upsampled high-level semantic features is beneficial for this task. The shallow and deep features have different response characteristics: shallow features preserve fine-grained edges and stroke details, while high-level features provide stronger semantic discrimination. A single gate imposes a coupled fusion ratio on both streams, whereas dual gates allow the two feature streams to be adjusted independently at each spatial location. Therefore, the dual-gate design provides more flexible feature recalibration and improves cross-scale fusion quality.

Comment 5:

The foreground mask in the manuscript is generated based on the probability map of the clean branch, and the threshold τ = 0.2 is an empirical value. The authors did not conduct a hyperparameter sensitivity analysis, so they cannot prove the universality of this threshold. Errors in the predictions of some clean branches can lead to an inaccurate foreground mask, which in turn causes the consistency loss to generate erroneous supervision signals.

Response to Comment 5:

Thank you for pointing this out. In the revised manuscript, we added a hyperparameter sensitivity analysis for the region-weighted consistency learning strategy. Specifically, we varied the foreground threshold τ, the foreground weight α, and the background weight β while keeping the other parameters fixed. The results show that τ = 0.20, α = 3, and β = 0.3 provide a favorable balance between Precision, Recall, and F1 score. For τ, the model maintains stable performance within a reasonable range, and τ = 0.20 achieves a good balance between text-region coverage and false-positive suppression.

We also clarified that the mask generated from the clean branch is not used as a hard pseudo-label to replace ground-truth supervision. Instead, it is used only to construct a region-weight map for the consistency loss. The main detection loss is still supervised by manual annotations. In addition, the background weight is not set to zero, and the consistency loss is assigned a relatively small coefficient in the total loss. These designs reduce the potential negative influence of inaccurate clean-branch predictions and prevent erroneous foreground masks from dominating the training process.

Comment 6:

The manuscript does not compare the proposed method with the latest lightweight text detection models, such as DBNet++, the MobileNetV3 backbone, YOLOv8/YOLOv9-based models, or the detection components of PP-OCRv3/v4. These models may offer advantages in terms of accuracy and speed.

Response to Comment 6:

Thank you for this constructive suggestion. We agree that comparisons with more recent and representative lightweight text detection methods can further strengthen the persuasiveness of the experimental results. In the original manuscript, we had already included comparisons with DBNet++ and different lightweight backbones, including ShuffleNetV2, EfficientNet, MobileNetV3, and MobileNetV4. Following the reviewer’s suggestion, we further expanded the comparison in the revised manuscript by adding PP-OCRv4 and a YOLO-series detector on the self-collected surgical instrument code dataset.

All compared methods were evaluated under the same data split, input resolution, evaluation metrics, and hardware platform. The experimental results show that LA-DBNet achieves the highest F1 score on the self-collected dataset, while maintaining a smaller model size and favorable inference efficiency compared with most conventional text detection methods. These additional comparisons further validate the effectiveness and lightweight advantage of the proposed method for surgical instrument code detection.

Comment 7:

The manuscript’s ablation experiments only verify the overall contributions of major modules and do not perform fine-grained ablation on key components within the modules. For example, what are the individual contributions of the direction enhancement, difference prompt, and dual-gating components within the DECA module? How much would removing any one of these components affect performance?

Response to Comment 7:

Thank you for this constructive comment. We agree that the ablation experiments in the original manuscript mainly verified the overall contribution of major modules, while the individual effects of key components within DECA were not sufficiently analyzed. Following the reviewer’s suggestion, we added a fine-grained ablation study of DECA in the revised manuscript, separately evaluating the effects of Directional Enhancement, Discrepancy Prompt, the discrepancy measurement strategy, and Dual Gates.

The results show that Full DECA achieved the highest F1 of 93.8%. Removing Directional Enhancement reduced F1 to 91.4%, indicating that directional enhancement helps capture the edges and stroke structures of elongated engraved characters. Removing the Discrepancy Prompt reduced F1 to 92.6%, and Precision decreased from 98.1% to 94.3%, suggesting that the discrepancy cue helps alleviate local response inconsistencies during cross-scale fusion and suppress false positives. Replacing absolute difference with cosine distance resulted in an F1 of 93.4%, slightly lower than that of Full DECA, indicating that absolute difference is more suitable for capturing local response discrepancies between adjacent-scale features in this task. Replacing Dual Gates with a Single Gate decreased F1 to 92.8%, showing that the dual-gate mechanism can more effectively recalibrate shallow detail features and high-level semantic features separately.

These results further validate the effectiveness of the key components within DECA. Among them, removing Directional Enhancement caused the most pronounced decrease in F1 and Recall, indicating that directional enhancement is particularly important for detecting elongated engraved codes. The corresponding experiments and analysis have been added to the fine-grained DECA ablation section of the revised manuscript.

Comment 8:

The manuscript mentions that the dataset is split at the instance level, but does not specify the exact splitting criteria or process. For example, is the split based on instrument instances or categories? How many images are there per instance? Does the splitting ensure category balance between the training and test sets?

Response to Comment 8:

Thank you for this careful comment. We have further clarified the dataset splitting strategy in the revised manuscript. The split was performed at the physical instrument instance level rather than at the image level. In our dataset, each physical instrument was captured under different viewpoints and imaging conditions, with approximately 10–20 images collected for one instrument. All images from the same physical instrument were assigned exclusively to one subset, so that visually similar images of the same instrument would not appear across the training, validation, and test sets. Meanwhile, we attempted to maintain approximately balanced category distributions among the three subsets. This clarification has been added to the dataset description section of the revised manuscript.

Comment 9:

The manuscript states that the default training and post-processing settings from the official implementation were retained, but no model-specific tuning was performed for the surgical instrument code dataset. Since LA-DBNet was specifically designed for this task, is this comparison method fair to other models?

Response to Comment 9:

Thank you for raising this important concern. We agree that the fairness of the comparative experiments should be further clarified. In the original manuscript, we had a

Attachment Submitted filename: Response to Reviewers.docx

# Peer review: Decision Letter 1

**DOI:** 10.1371/journal.pone.0355611.r003

**Authors:** Athanasios Pantelis

13 Jul 2026

PONE-D-26-18770R1

A lightweight alignment-aware DBNet for surgical instrument code detection

PLOS One

Dear Dr. Qu,

Thank you for submitting your manuscript to PLOS ONE. After careful consideration, we feel that it has merit but does not fully meet PLOS ONE’s publication criteria as it currently stands. Therefore, we invite you to submit a revised version of the manuscript that addresses the points raised during the review process.

==============================

**ACADEMIC EDITOR:**  Please see my comments below.

==============================

Please submit your revised manuscript by Aug 27 2026 11:59PM If you will need more time than this to complete your revisions, please reply to this message or contact the journal office at plosone@plos.org. When you're ready to submit your revision, log on to https://www.editorialmanager.com/pone/ and select the 'Submissions Needing Revision' folder to locate your manuscript file.

Please include the following items when submitting your revised manuscript:

- A letter that responds to each point raised by the academic editor and reviewer(s). You should upload this letter as a separate file labeled 'Response to Reviewers'.
- A marked-up copy of your manuscript that highlights changes made to the original version. You should upload this as a separate file labeled 'Revised Manuscript with Track Changes'.
- An unmarked version of your revised paper without tracked changes. You should upload this as a separate file labeled 'Manuscript'.

If you would like to make changes to your financial disclosure, please include your updated statement in your cover letter. Guidelines for resubmitting your figure files are available below the reviewer comments at the end of this letter.

If applicable, we recommend that you deposit your laboratory protocols in protocols.io to enhance the reproducibility of your results. Protocols.io assigns your protocol its own identifier (DOI) so that it can be cited independently in the future. For instructions see: https://journals.plos.org/plosone/s/submission-guidelines#loc-laboratory-protocols. Additionally, PLOS ONE offers an option for publishing peer-reviewed Lab Protocol articles, which describe protocols hosted on protocols.io. Read more information on sharing protocols at https://plos.org/protocols?utm_medium=editorial-email&utm_source=authorletters&utm_campaign=protocols.

As the corresponding author, your ORCID iD is verified in the submission system and will appear in the published article. PLOS supports the use of ORCID, and we encourage all coauthors to register for an ORCID iD and use it as well. Please encourage your coauthors to verify their ORCID iD within the submission system before final acceptance, as unverified ORCID iDs will not appear in the published article. *Only*  the individual author can complete the verification step; PLOS staff *cannot*  verify ORCID iDs on behalf of authors.

We look forward to receiving your revised manuscript.

Kind regards,

Athanasios G. Pantelis

Academic Editor

PLOS One

Journal Requirements:

If the reviewer comments include a recommendation to cite specific previously published works, please review and evaluate these publications to determine whether they are relevant and should be cited. There is no requirement to cite these works unless the editor has indicated otherwise.

Please review your reference list to ensure that it is complete and correct. If you have cited papers that have been retracted, please include the rationale for doing so in the manuscript text, or remove these references and replace them with relevant current references. Any changes to the reference list should be mentioned in the rebuttal letter that accompanies your revised manuscript. If you need to cite a retracted article, indicate the article’s retracted status in the References list and also include a citation and full reference for the retraction notice.

Additional Editor Comments (if provided):

Dear Authors,

Thank you for submitting your revised manuscript. I appreciate the considerable effort invested in addressing the reviewers' comments. The manuscript has improved substantially, and the additional experiments and clarifications have strengthened the work.

Before I can recommend acceptance, I would appreciate if you could address the following minor issues, in addition to the reviewrs' comments:

1) Data and code availability: Please ensure that the Data Availability Statement complies with PLOS ONE policy. Clarify whether the dataset, annotations, trained model weights, and/or source code are publicly available. If any of these cannot be shared, please provide an appropriate justification.

2) Statistical robustness: Please clarify whether the reported results are based on a single training run or multiple independent runs. If only a single run was performed, please acknowledge this as a limitation.

3) Generalizability: Please moderate statements suggesting broad generalizability, as the proposed method has been validated primarily on a single proprietary surgical dataset and one public benchmark.

4) Evaluation protocol: Please provide a brief clarification that all comparative methods were evaluated under identical experimental conditions and specify any factors that may affect the reported inference speed (e.g., framework, implementation, and hardware).

I believe these are relatively minor points, and I look forward to reviewing the revised version.

[Note: HTML markup is below. Please do not edit.]

Reviewers' comments:

Reviewer's Responses to Questions

**Comments to the Author**

1. If the authors have adequately addressed your comments raised in a previous round of review and you feel that this manuscript is now acceptable for publication, you may indicate that here to bypass the “Comments to the Author” section, enter your conflict of interest statement in the “Confidential to Editor” section, and submit your "Accept" recommendation.

Reviewer #1: (No Response)

Reviewer #2: All comments have been addressed

**********

2. Is the manuscript technically sound, and do the data support the conclusions?

The manuscript must describe a technically sound piece of scientific research with data that supports the conclusions. Experiments must have been conducted rigorously, with appropriate controls, replication, and sample sizes. The conclusions must be drawn appropriately based on the data presented.

Reviewer #1: (No Response)

Reviewer #2: Yes

**********

3. Has the statistical analysis been performed appropriately and rigorously?

Reviewer #1: (No Response)

Reviewer #2: Yes

**********

4. Have the authors made all data underlying the findings in their manuscript fully available?

The PLOS Data policy requires authors to make all data underlying the findings described in their manuscript fully available without restriction, with rare exception (please refer to the Data Availability Statement in the manuscript PDF file). The data should be provided as part of the manuscript or its supporting information, or deposited to a public repository. For example, in addition to summary statistics, the data points behind means, medians and variance measures should be available. If there are restrictions on publicly sharing data—e.g. participant privacy or use of data from a third party—those must be specified.

Reviewer #1: (No Response)

Reviewer #2: Yes

**********

5. Is the manuscript presented in an intelligible fashion and written in standard English?

PLOS ONE does not copyedit accepted manuscripts, so the language in submitted articles must be clear, correct, and unambiguous. Any typographical or grammatical errors should be corrected at revision, so please note any specific errors here.

Reviewer #1: (No Response)

Reviewer #2: Yes

**********

6. Review Comments to the Author

Please use the space provided to explain your answers to the questions above. You may also include additional comments for the author, including concerns about dual publication, research ethics, or publication ethics. (Please upload your review as an attachment if it exceeds 20,000 characters)

Reviewer #1: (No Response)

Reviewer #2: (No Response)

**********

7. PLOS authors have the option to publish the peer review history of their article (what does this mean?). If published, this will include your full peer review and any attached files.

If you choose “no”, your identity will remain anonymous but your review may still be made public.

**Do you want your identity to be public for this peer review?**  For information about this choice, including consent withdrawal, please see our Privacy Policy.

Reviewer #1: No

Reviewer #2: No

**********

[NOTE: If reviewer comments were submitted as an attachment file, they will be attached to this email and accessible via the submission site. Please log into your account, locate the manuscript record, and check for the action link "View Attachments". If this link does not appear, there are no attachment files.]

To ensure your figures meet our technical requirements, please review our figure guidelines: https://journals.plos.org/plosone/s/figures

You may also use PLOS’s free figure tool, NAAS, to help you prepare publication quality figures: https://journals.plos.org/plosone/s/figures#loc-tools-for-figure-preparation.

NAAS will assess whether your figures meet our technical requirements by comparing each figure against our figure specifications.

# Author comment: Author response to Decision Letter 2

**DOI:** 10.1371/journal.pone.0355611.r004

13 Jul 2026

Manuscript number: PONE-D-26-18770R1

Title: A lightweight alignment-aware DBNet for surgical instrument code detection

Dear Editor and Reviewers,

We sincerely thank you for your careful evaluation of our manuscript and for your thoughtful and constructive comments. We greatly appreciate the time and effort you have devoted to reviewing our work. In response to the comments and suggestions provided, we have carefully re-examined the entire manuscript and revised the relevant sections in accordance with the journal’s requirements. We have also conducted a thorough check of the manuscript to improve its clarity, accuracy, transparency, and overall presentation. All changes have been clearly marked in the revised manuscript, and our detailed point-by-point responses are provided below.

Editor Comment 1:

Data and code availability: Please ensure that the Data Availability Statement complies with PLOS ONE policy. Clarify whether the dataset, annotations, trained model weights, and/or source code are publicly available. If any of these cannot be shared, please provide an appropriate justification.

Response to Comment 1:

Thank you for this important comment. We have revised the Data Availability Statement to clearly describe the availability of all research materials associated with this study. The datasets, annotation files, trained model weights, and source code used in this work are publicly available without restriction through our GitHub repository at: https://github.com/wy49497/LADBNet.

Editor Comment 2:

Statistical robustness: Please clarify whether the reported results are based on a single training run or multiple independent runs. If only a single run was performed, please acknowledge this as a limitation.

Response to Comment 2:

Thank you for raising this important point. We have clarified in the revised manuscript that the reported results were obtained from a single independent training and evaluation run for each model. Therefore, the reported values represent the performance observed in that individual run rather than the mean performance across multiple runs with different random seeds.

We acknowledge that a single run does not capture the potential variability arising from random initialization, stochastic optimization, and data sampling. We have therefore explicitly added this issue as a limitation in the Discussion section. In future work, we will conduct multiple independent experiments using different random seeds and report the results as the mean and standard deviation, or with appropriate confidence intervals, to provide a more comprehensive assessment of statistical robustness.

Editor Comment 3:

Generalizability: Please moderate statements suggesting broad generalizability, as the proposed method has been validated primarily on a single proprietary surgical dataset and one public benchmark.

Response to Comment 3:

Thank you very much for this valuable comment. We agree that the generalizability of the proposed method should be interpreted with caution, since the current experimental evaluation was conducted primarily on one self-collected surgical instrument dataset and one public benchmark dataset. Accordingly, we have carefully reviewed the entire manuscript and appropriately moderated the relevant statements, particularly in the Datasets section and Conclusion, to avoid implying broad or universal generalizability.

Editor Comment 4:

Evaluation protocol: Please provide a brief clarification that all comparative methods were evaluated under identical experimental conditions and specify any factors that may affect the reported inference speed (e.g., framework, implementation, and hardware).

Response to Comment 4:

Thank you very much for this important comment. We have clarified the evaluation protocol in the revised manuscript. All experiments conducted within each dataset adopted identical data splits, input resolutions, evaluation metrics, test hardware platforms, and evaluation protocols. For methods with publicly available implementations, their official training and post-processing settings were retained as much as possible.

We have also clarified that the reported inference speed was measured under the experimental environment listed in Table 1 and may be affected by factors such as the deep-learning framework, code implementation and optimization strategy, hardware configuration, batch size, and input resolution.

We sincerely appreciate the Academic Editor’s constructive comments, which have helped us further improve the clarity, transparency, and rigor of the manuscript.

Sincerely,

The Authors

Attachment Submitted filename: Response_to_Reviewers_auresp_2.docx

# Editor report: Decision Letter 2

**DOI:** 10.1371/journal.pone.0355611.r005

**Authors:** Athanasios Pantelis

20 Jul 2026

PONE-D-26-18770R2

A lightweight alignment-aware DBNet for surgical instrument code detection

PLOS One

Dear Dr. Qu,

Thank you for submitting your manuscript to PLOS ONE. After careful consideration, we feel that it has merit but does not fully meet PLOS ONE’s publication criteria as it currently stands. Therefore, we invite you to submit a revised version of the manuscript that addresses the points raised during the review process.

==============================

**ACADEMIC EDITOR: Please provide a clear data availabity statement.**

==============================

Please submit your revised manuscript by  Sep 03 2026 11:59PM. If you will need more time than this to complete your revisions, please reply to this message or contact the journal office at plosone@plos.org. When you're ready to submit your revision, log on to https://www.editorialmanager.com/pone/ and select the 'Submissions Needing Revision' folder to locate your manuscript file.

Please include the following items when submitting your revised manuscript:

- A letter that responds to each point raised by the academic editor and reviewer(s). You should upload this letter as a separate file labeled 'Response to Reviewers'.
- A marked-up copy of your manuscript that highlights changes made to the original version. You should upload this as a separate file labeled 'Revised Manuscript with Track Changes'.
- An unmarked version of your revised paper without tracked changes. You should upload this as a separate file labeled 'Manuscript'.

If you would like to make changes to your financial disclosure, please include your updated statement in your cover letter. Guidelines for resubmitting your figure files are available below the reviewer comments at the end of this letter.

If applicable, we recommend that you deposit your laboratory protocols in protocols.io to enhance the reproducibility of your results. Protocols.io assigns your protocol its own identifier (DOI) so that it can be cited independently in the future. For instructions see: https://journals.plos.org/plosone/s/submission-guidelines#loc-laboratory-protocols. Additionally, PLOS ONE offers an option for publishing peer-reviewed Lab Protocol articles, which describe protocols hosted on protocols.io. Read more information on sharing protocols at https://plos.org/protocols?utm_medium=editorial-email&utm_source=authorletters&utm_campaign=protocols.

As the corresponding author, your ORCID iD is verified in the submission system and will appear in the published article. PLOS supports the use of ORCID, and we encourage all coauthors to register for an ORCID iD and use it as well. Please encourage your coauthors to verify their ORCID iD within the submission system before final acceptance, as unverified ORCID iDs will not appear in the published article. *Only*  the individual author can complete the verification step; PLOS staff *cannot*  verify ORCID iDs on behalf of authors.

We look forward to receiving your revised manuscript.

Kind regards,

Athanasios G. Pantelis

Academic Editor

PLOS One

Journal Requirements:

If the reviewer comments include a recommendation to cite specific previously published works, please review and evaluate these publications to determine whether they are relevant and should be cited. There is no requirement to cite these works unless the editor has indicated otherwise.

Please review your reference list to ensure that it is complete and correct. If you have cited papers that have been retracted, please include the rationale for doing so in the manuscript text, or remove these references and replace them with relevant current references. Any changes to the reference list should be mentioned in the rebuttal letter that accompanies your revised manuscript. If you need to cite a retracted article, indicate the article’s retracted status in the References list and also include a citation and full reference for the retraction notice.

Additional Editor Comments:

Dear Dr. Qu,

Thank you for your careful revision of manuscript PONE-D-26-18770R1 and for the detailed point-by-point response. I have reviewed the revised manuscript against your responses to the four prior comments.

I'm pleased to confirm that Comments 2 (statistical robustness), 3 (generalizability), and 4 (evaluation protocol) have been satisfactorily addressed in the revised text.

However, before I can proceed with acceptance of your manuscript, the following item needs to be resolved:

Data Availability Statement (Comment 1): Your response states that the Data Availability Statement has been revised to reference your GitHub repository, but I was unable to locate this statement anywhere in the manuscript file. Please add the Data Availability Statement directly to the manuscript (not only the submission system field), and confirm that the repository is public and contains the dataset, annotations, trained model weights, and source code as described.

I look forward to receiving the completed manuscript.

Athanasios Pantelis, MD, MSc, FMBS, FACS

General and Bariatric Surgeon

Academic Editor, PLOS ONE

[Note: HTML markup is below. Please do not edit.]

[NOTE: If reviewer comments were submitted as an attachment file, they will be attached to this email and accessible via the submission site. Please log into your account, locate the manuscript record, and check for the action link "View Attachments". If this link does not appear, there are no attachment files.]

To ensure your figures meet our technical requirements, please review our figure guidelines: https://journals.plos.org/plosone/s/figures

You may also use PLOS’s free figure tool, NAAS, to help you prepare publication quality figures: https://journals.plos.org/plosone/s/figures#loc-tools-for-figure-preparation.

NAAS will assess whether your figures meet our technical requirements by comparing each figure against our figure specifications.

# Author comment: Author response to Decision Letter 3

**DOI:** 10.1371/journal.pone.0355611.r006

20 Jul 2026

Manuscript number: PONE-D-26-18770R2

Title: A lightweight alignment-aware DBNet for surgical instrument code detection

Dear Editor,

Thank you for your careful evaluation of our revised manuscript and for bringing this omission to our attention. We sincerely apologise that the Data Availability Statement was inadvertently omitted from the previous revised manuscript file, although the relevant information had been entered in the submission system.

In response to your comment, we have now added a separate “Data Availability Statement” directly before the References section of the revised manuscript.

We have also verified that the research materials are publicly accessible. The image dataset has been deposited in Zenodo and is publicly available at https://doi.org/10.5281/zenodo.21452216. The annotation files, trained model weights, source code, and corresponding usage instructions are publicly available in the GitHub repository at https://github.com/wy49497/LADBNet.

Editor Comment 1:

Data Availability Statement (Comment 1): Your response states that the Data Availability Statement has been revised to reference your GitHub repository, but I was unable to locate this statement anywhere in the manuscript file. Please add the Data Availability Statement directly to the manuscript (not only the submission system field), and confirm that the repository is public and contains the dataset, annotations, trained model weights, and source code as described.

Response to Comment 1:

Thank you for pointing this out. We sincerely apologise for the omission of the Data Availability Statement from the previous revised manuscript file. Although the relevant information had been provided in the submission system, the statement was inadvertently omitted from the manuscript itself.

We have now added a separate “Data Availability Statement” directly before the References section of the revised manuscript. We confirm that the image dataset has been deposited in Zenodo and is publicly accessible at https://doi.org/10.5281/zenodo.21452216. The annotation files, trained model weights, source code, and corresponding usage instructions are publicly accessible through our GitHub repository at https://github.com/wy49497/LADBNet.

The following statement has been added to the manuscript:

“Data Availability Statement: The image dataset underlying the findings of this study is publicly available in the Zenodo repository at https://doi.org/10.5281/zenodo.21452216. The annotation files, trained model weights, source code, and instructions for accessing and using these resources are publicly available in the GitHub repository at https://github.com/wy49497/LADBNet.”

Thank you for bringing this omission to our attention. We have uploaded the corrected manuscript accordingly.

Sincerely,

The Authors

Attachment Submitted filename: Response_to_Reviewers_auresp_3.docx

# Editor report: Decision Letter 3

**DOI:** 10.1371/journal.pone.0355611.r007

**Authors:** Athanasios Pantelis

23 Jul 2026

A lightweight alignment-aware DBNet for surgical instrument code detection

PONE-D-26-18770R3

Dear Dr. Qu,

We’re pleased to inform you that your manuscript has been judged scientifically suitable for publication and will be formally accepted for publication once it meets all outstanding technical requirements.

Within one week, you’ll receive an e-mail detailing the required amendments. When these have been addressed, you’ll receive a formal acceptance letter and your manuscript will be scheduled for publication.

An invoice will be generated when your article is formally accepted. Please note, if your institution has a publishing partnership with PLOS and your article meets the relevant criteria, all or part of your publication costs will be covered. Please make sure your user information is up-to-date by logging into Editorial Manager at Editorial Manager® and clicking the ‘Update My Information' link at the top of the page. For questions related to billing, please contact billing support.

If your institution or institutions have a press office, please notify them about your upcoming paper to help maximize its impact. If they’ll be preparing press materials, please inform our press team as soon as possible -- no later than 48 hours after receiving the formal acceptance. Your manuscript will remain under strict press embargo until 2 pm Eastern Time on the date of publication. For more information, please contact onepress@plos.org.

Kind regards,

Athanasios G. Pantelis

Academic Editor

PLOS One

Additional Editor Comments (optional):

All reviewers' comments have adequately and effectively been addressed. Congratulations to the authors for their original research.

Reviewers' comments:

# Editor report: Acceptance letter

**DOI:** 10.1371/journal.pone.0355611.r008

**Authors:** Athanasios Pantelis

PONE-D-26-18770R3

PLOS One

Dear Dr. Qu,

I'm pleased to inform you that your manuscript has been deemed suitable for publication in PLOS One. Congratulations! Your manuscript is now being handed over to our production team.

At this stage, our production department will prepare your paper for publication. This includes ensuring the following:

* All references, tables, and figures are properly cited

* All relevant supporting information is included in the manuscript submission,

* There are no issues that prevent the paper from being properly typeset

You will receive further instructions from the production team, including instructions on how to review your proof when it is ready. Please keep in mind that we are working through a large volume of accepted articles, so please give us a few days to review your paper and let you know the next and final steps.

Lastly, if your institution or institutions have a press office, please let them know about your upcoming paper now to help maximize its impact. If they'll be preparing press materials, please inform our press team within the next 48 hours. Your manuscript will remain under strict press embargo until 2 pm Eastern Time on the date of publication. For more information, please contact onepress@plos.org.

You will receive an invoice from PLOS for your publication fee after your manuscript has reached the completed accept phase. If you receive an email requesting payment before acceptance or for any other service, this may be a phishing scheme. Learn how to identify phishing emails and protect your accounts at https://explore.plos.org/phishing.

If we can help with anything else, please email us at customercare@plos.org.

Thank you for submitting your work to PLOS ONE and supporting open access.

Kind regards,

PLOS ONE Editorial Office Staff

on behalf of

Dr. Athanasios G. Pantelis

Academic Editor

PLOS One
